# Ion sieving by a two-dimensional Ti_3_C_2_T_*x*_ alginate lamellar membrane with stable interlayer spacing

**DOI:** 10.1038/s41467-020-17373-4

**Published:** 2020-07-15

**Authors:** Jin Wang, Zhijie Zhang, Jiani Zhu, Mengtao Tian, Shuchang Zheng, Fudi Wang, Xudong Wang, Lei Wang

**Affiliations:** Research Institute of Membrane Separation Technology of Shaanxi Province, School of Environmental and Municipal Engineering, Xi’an University of Architecture and Technology, Xi’an, 710000 China

**Keywords:** Environmental chemistry, Two-dimensional materials, Water resources

## Abstract

Two-dimensional membranes attract extensive interest due to the anomalous transport phenomena; however, the ion separation performance is below the theoretical prediction. The stabilization of d-spacing is a key step for enhancing ion selectivity. Here, we demonstrate a strategy for stabilizing the Ti_3_C_2_T_*x*_ laminar architecture by alginate hydrogel pillars. After pillared by Ca-alginate, the nanochannel diameters are effectively fixed at 7.4 ± 0.2 Å, and the membrane presents a permeation cutoff and an outstanding sieving property towards valent cations. When applied for acid recovery, the outstanding H^+^/Fe^2+^ selectivity makes the membrane a promising substitution for traditional ion-exchange membranes. Moreover, the ultrathin Mn-alginate pillared membrane with identical d-spacing exhibits 100% Na_2_SO_4_ rejection with high water permeance, which is superior to the state-of-the-art nanofiltration membranes. Building on these findings, we demonstrate an efficient method to tune the ion selectivity and introduce a new perspective for energy- and environment-related applications.

## Abstract (web summary)

Two dimensional lamellar membranes are attractive for anomalous water and ion transfer, but performance is hindered by swelling. Here, the authors stabilize a MXene membrane laminar architecture with fixed nanochannels, achieving highly selective acid recovery from iron-based wastewater.

## Introduction

The considerable progress made during the last decade in ultrathin two-dimensional (2D) materials has inspired research on fluid behavior at the nanometer scale and offers a new perspective for technological breakthroughs in a variety of applications, including ion sieving, molecular separation, and desalination^1–5^. The separation membranes derived from a single layer of 2D materials with uniform nanopores exhibit an extraordinary molecular separation performance, which is attributed to the atom-thick membrane that minimizes the transport resistance and subsequently maximizes the permeate flux^6,7^. However, the fabrication of a large-area integrated nanosheet with abundant angstrom-precise pores and effective approaches for scaling atom-thick membranes into applicable devices remain obstacles to achieve the challenging goals^8^. Membranes with a laminar architecture assembled by parallel restacking of monolayer nanosheets represent a more practical option than other membrane configurations for generating highly ordered nanoscale channels, and they have attracted increasing research interest^9,10^. Demands for 2D lamellar membranes with exceptional performance have stimulated research on novel 2D materials and synthetic methods, membrane fabrication technology, liquid transport mechanisms in extremely confined areas, etc.^11–13^.

To date, 2D nanomaterials for laminar membrane fabrication are primarily based on graphene oxide (GO) and other graphene derivatives^1–4,14,15^, and recently, various nanomaterials, such as hexagonal boron nitride, transition metal dichalcogenides, and layered double hydroxides, have been proposed as promising candidates for membrane assembly^16–19^. MXene is a fast-growing family of 2D layered transition metal carbides and/or nitrides with the general formula of M_*n*+1_X_*n*_T_*x*_ (*n* = 1, 2, 3, or 4), where M represents one or more early transition metals, X represents carbon and/or nitrogen, and T_*x*_ represents surface groups (–O,–OH, and/or –F)^20–22^. As the most studied MXene phase, 2D titanium carbide, Ti_3_C_2_T_*x*_, exhibits excellent mechanical properties and structural stability in aqueous solution, which is critical for GO membranes in solution-based applications^23,24^. In addition, the functionalized surface allows Ti_3_C_2_T_*x*_ to bond effectively with various species^25,26^. Ti_3_C_2_T_*x*_ laminates have been evaluated as a potential competitor in energy storage, electromagnetic interference shielding, biosensors and other fields^27–30^, and their activity in ion sieving has recently begun to be described^31,32^.

The transport of molecules and ions in 2D lamellar membranes is considered to occur in the massively interconnected capillaries formed between two neighboring stacked nanosheets; therefore, the diameter of the capillary (also known as the interlayer spacing or d-spacing) significantly influences the membrane selectivity and separation properties^33,34^. Many studies have confirmed that the anomalous transport phenomena in laminar membranes, such as ultrafast and precise ion selectivity, could be facilitated when the d-spacing is comparable with the diameter *D* of hydrated ions^12,35,36^, However, the actual separation performance of 2D laminates in aqueous solution is still far from the theoretically predicted performance, which could be because the oxygen-containing surface functional groups on both the GO and MXene planes tend to induce membrane swelling and result in expansion of the d-spacing^23,37,38^. Ren et al. suggested that the Ti_3_C_2_T_*x*_ membranes show selectivity towards cations with different charges, whereas the membranes do not exhibit a permeation cutoff similar to the GO membrane because cations from aqueous solutions could readily intercalate into Ti_3_C_2_T_*x*_ laminates^31^. Accordingly, inhibiting the swelling effect and stably constraining the angstrom-scale interlayer are considered keys to enhancing the selectivity property of 2D lamellar membranes. A number of alternative strategies have been proposed to reduce the interlayer spacing, such as physical confinement, cation intercalation and self-cross-linking, leading to a high rejection of ions, independent of size or valence^12,32,39,40^. These membranes demonstrated the exclusion ability to the monovalent ions and hold the promise for desalination. Several studies have also been conducted to enhance the membrane structure stability by covalent cross-linking by polymer molecules; however, due to the volume effect induced by the cross-linking reactions, the membranes showed an improved molecular separation property but a decreased selectivity towards ions^41–44^.

**Fig. 1:**
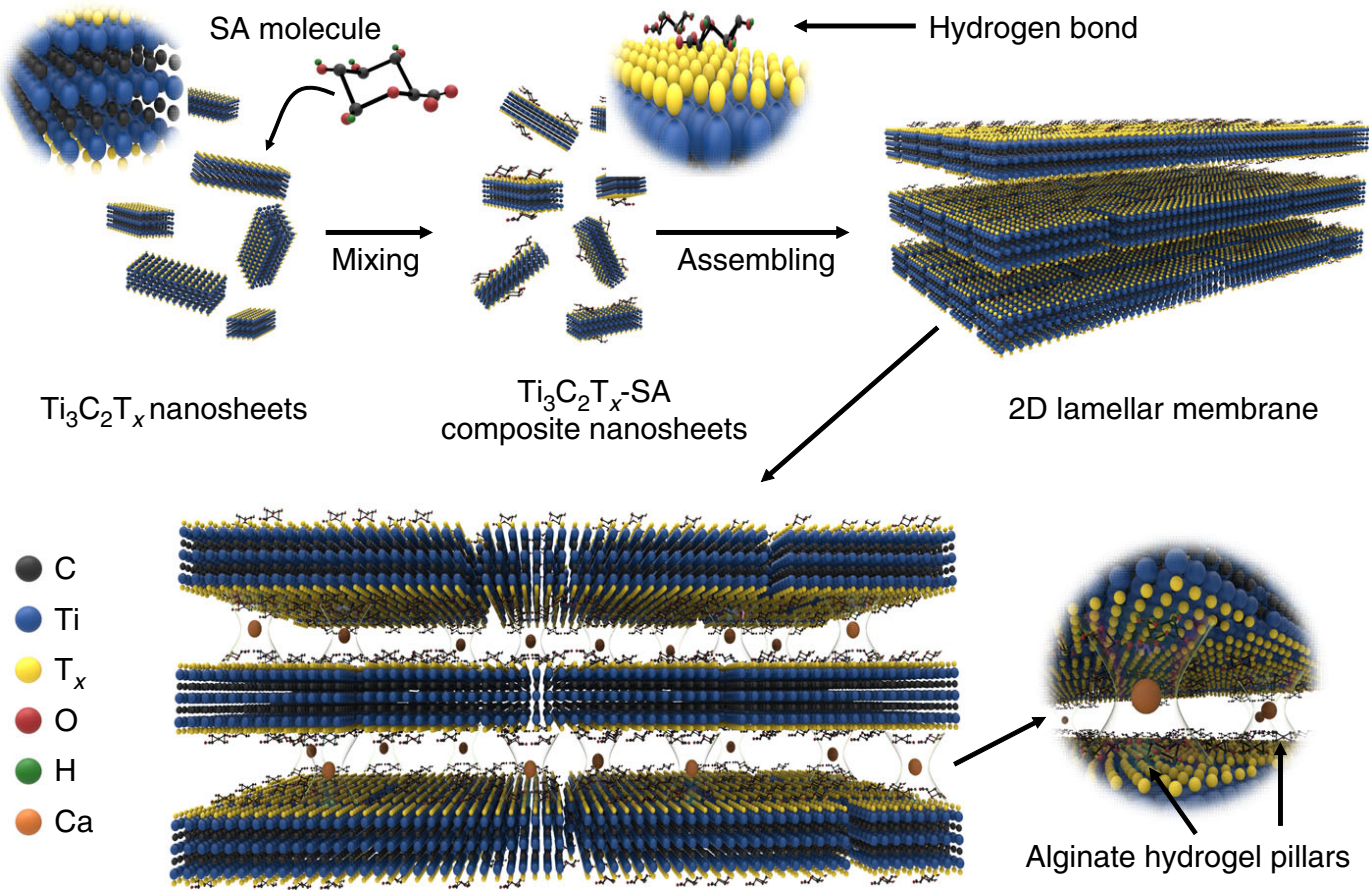
Schematic illustration of the preparation of 2D lamellar Ti_3_C_2_T_*x*_ alginate membranes. After sodium alginate (SA) solution was mixed with the Ti_3_C_2_T_*x*_ colloidal solution, the SA molecules were firmly and homogeneously attached onto the nanosheet surface via hydrogen bonding. Then, the composite SA-Ti_3_C_2_T_*x*_ nanosheets were assembled into a hybrid SA-Ti_3_C_2_T_*x*_ membrane with a lamellar structure. Finally, the SA-Ti_3_C_2_T_*x*_ membrane was immersed into different multivalent M^n+^ cross-linking solutions (Ca^2+^, Ba^2+^, Mn^2+^, and Al^3+^) to obtain the cross-linked membrane with hydrogel pillars in the interlayer spacing.

In this paper, we demonstrate a methodology for stabilizing the Ti_3_C_2_T_*x*_ membrane laminar architecture via alginate hydrogel pillars formed in the interlayer spacing. The sodium alginate (SA) molecules are first homogeneously anchored on the Ti_3_C_2_T_*x*_ nanosheet surface, and after assembly by van der Waals forces, the SA-Ti_3_C_2_T_*x*_ membrane is immersed into multivalent cation solutions to complete the cross-linking reaction. The pillared Ti_3_C_2_T_*x*_ membranes (termed the M-SAT membrane, where M represents Ca, Ba, Mn, and Al) with a uniform nacre-like structure could significantly restrict the swelling effect (Fig. 1). Although the effective diameters of nanochannels with different alginate spacers are all maintained at ~7.4 ± 0.2 Å, the membranes exhibit different ion selectivities for the valences of cations or anions.

## Results

### Characterizations of nanosheets and M-SAT membranes

**Fig. 2:**
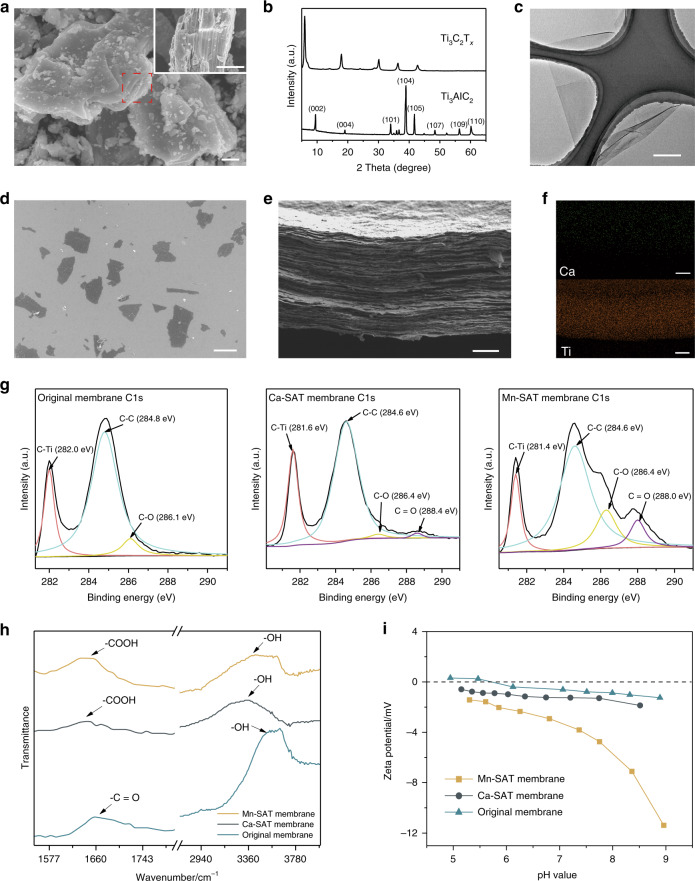
Characterizations of Ti_3_C_2_T_*x*_ nanosheets and Ca-SAT membranes. **a** SEM image of the MAX phase Ti_3_AlC_2_ powder (scale bar is 2 μm). **b** XRD patterns of the Ti_3_AlC_2_ powder and the Ti_3_C_2_T_*x*_ nanosheets. **c** TEM image of a monolayer Ti_3_C_2_T_*x*_ nanosheet (scale bar is 500 nm). **d** SEM images of the Ti_3_C_2_T_*x*_ nanosheets deposited on the silicon substrate (scale bar is 1 μm). **e** SEM image of a cross-section of the Ca-SAT membrane (scale bar is 1 μm). **f** EDS elemental maps of the Ca-SAT membrane (scale bar is 1 μm). **g** C 1*s* XPS spectra with the fitting results of the original and pillared Ti_3_C_2_T_*x*_ membrane. **h** FTIR spectra of the original and pillared membranes. **i** Surface zeta potentials of the original and pillared membranes.

We synthesized Ti_3_C_2_T_*x*_ by selectively etching the aluminum layers from the ternary carbide MAX phase precursor (Ti_3_AlC_2_) in aqueous LiF-HCl mixing solution. The selective removal of Al in the MAX phase is confirmed by the complete disappearance of the diffraction peak at 39° and the left shift of the (002) peak in the X-ray diffraction (XRD) spectrum of Ti_3_C_2_T_*x*_. The scanning electron microscopy (SEM) images (Fig. 2a, d) also indicate that Ti_3_AlC_2_ with a characteristic plate-like structure is exfoliated into a single layer of Ti_3_C_2_T_*x*_ 1–3 μm in lateral size. The transmission electron microscopy (TEM) image (Fig. 2c) reveals that the Ti_3_C_2_T_*x*_ nanosheet is quite thin and nearly transparent, with no structural defects. The Ti_3_C_2_T_*x*_ colloidal suspension shows a clear Tyndall scattering effect and typical UV–vis spectral characteristics (Supplementary Fig. 1). In the atomic force microscopy (AFM) image (Supplementary Fig. 2), the Ti_3_C_2_T_*x*_ nanosheets exhibit a uniform thickness of 1.2 nm. The high aspect ratio of the single-layer Ti_3_C_2_T_*x*_ ensures the uniformity of intercapillaries and mitigates the presence of meso- and macropores across the membrane. The SEM images of the surface and cross-section of the Ca-alginate pillared Ti_3_C_2_T_*x*_ membrane (Fig. 2e, Supplementary Figs. 3 and 4) demonstrate that the pillared membrane has a surface appearance and a cross-section identical to those of the as-synthesized Ti_3_C_2_T_*x*_ membrane^31,32^ and that the lamellar architecture is essentially unaffected by incorporation of the alginate gel spacer. The energy dispersive X-ray spectroscopy (EDS) elemental maps (Fig. 2f and Supplementary Fig. 3) of the Ca-SAT membrane show that the Ti, O, and cross-linking cation Ca^2+^ were distributed homogeneously throughout the cross-section. The Al originally contained in the MAX phase cannot be detected in the spectrum. In addition, similar to the as-synthesized Ti_3_C_2_T_*x*_ membrane, the pillared membrane presents impressive flexibility (Supplementary Fig. 5).

The chemical characterization of the original and pillared Ti_3_C_2_T_*x*_ membranes was confirmed by X-ray photoelectron spectroscopy (XPS) and Fourier transform infrared (FTIR) analysis (Fig. 2g, h and Supplementary Fig. 6). For the original membrane, the high-resolution XPS spectra profiles of O 1*s* are fitted with three peaks located at binding energies of 530.0 eV for TiO_2−*x*_F_*x*_, 531.1 eV for C–Ti–O_*x*_ and 531.9 eV for C–Ti–(OH)_x_. In the C 1*s* spectra, the fitted peak at a binding energy of 286.10 eV is attributed to C–O, and the peaks at 282.0 and 284.8 eV are assigned to C–Ti and C–C^45^. In the Mn-SAT membrane XPS spectra, in addition to the above typical peaks shown in the original membrane, both the C 1*s* peak at 288.0 eV and the O 1*s* peak at 533.3 eV verify the appearance of –COOH groups on the Mn-SAT membrane surface, whereas for the Ca-SAT membrane, the peaks that identify the –COOH groups are not evident. The FTIR spectra of the original Ti_3_C_2_T_*x*_ membrane present the characteristic peaks of –OH stretching at ~3400 cm^−1^ and C=O stretching at ~1680 cm^−1^. For the Mn-SAT membrane, the redshifts of the –OH peaks located at 3300 cm^−1^ confirm the formation of hydrogen bonds between Ti_3_C_2_T_*x*_ and SA molecules, which is consistent with the measurement result of the colloidal solution zeta potential (Supplementary Fig. 7)^46^, and the peak at 1630 cm^−1^ indicates the existence of carboxyl groups, which is consistent with the XPS results (Supplementary Fig. 8). The zeta potentials of the original and pillared Ti_3_C_2_T_*x*_ membranes at different pH values in 0.001 mol/L KCl solution (Fig. 2i) show that both the original and Ca-alginate pillared Ti_3_C_2_T_*x*_ membranes exhibited an approximately neutral surface, whereas the Mn-SAT membrane exhibited a lower zeta potential, which varied from −11.8 to −1 mV without an isoelectric point.

### Interlayer spacing control of the M-SAT membrane

**Fig. 3:**
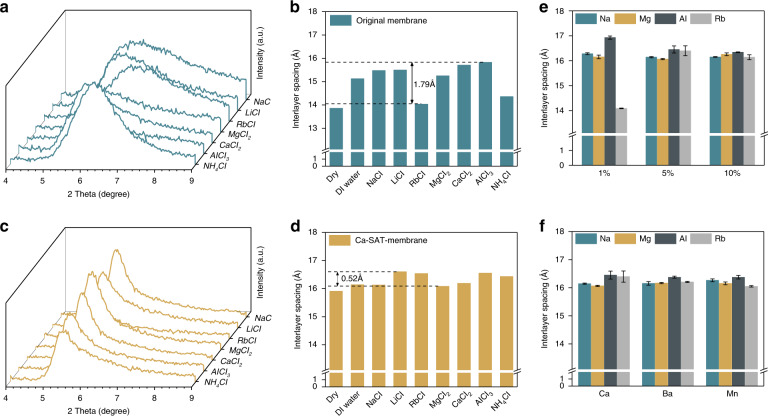
Interlayer spacings of original and pillared Ti_3_C_2_T_*x*_ membranes. **a**, **b** Interlayer spacings of the original membrane immersed in pure water or in various 0.5 mol L^−1^ salt solutions, as shown by the XRD pattern and bar chart. **c**, **d** Interlayer spacings of the Ca-SAT membrane immersed in pure water or in various 0.5 mol L^−1^ salt solutions, as shown in the bar chart and XRD pattern. **e** Interlayer spacings of the SA-Ti_3_C_2_T_*x*_ membrane cross-linked by Ca^2+^ with different amounts of sodium alginate (1%, 5%, and 10%). **f** Interlayer spacings of the SA-Ti_3_C_2_T_*x*_ membrane cross-linked by Ca^2+^, Ba^2+^, and Mn^2+^ immersed in various salt solutions. The error bars represent the standard deviations of three parallel tests.

The structural characteristics of the laminar Ti_3_C_2_T_*x*_ membrane before and after pillaring by alginate gel were confirmed via XRD analysis. For the original Ti_3_C_2_T_*x*_ membrane (Fig. 3a, b), the interlayer spacing demonstrates noticeable shifts after immersion in pure water and different chloride salt solutions. After soaking in water, the d-spacing of the pristine Ti_3_C_2_T_*x*_ membrane changed from 13.8 to 15.2 Å, which is consistent with the previous literature^47^. The structural changes become more complicated in different salt solutions in that the intercalation of most cations causes expansion of the interlayer spacing, whereas Rb^+^ and NH_4_^+^ resulted in a contraction of the layer architecture. Previous reports have confirmed that cations (and not anions) from aqueous solutions could spontaneously intercalate into the lamellar MXene membrane^37,38^, and the structural change depends on both the electrostatic attraction between the negatively charged Ti_3_C_2_T_*x*_ sheets and the intercalated cations as well as the steric effect induced by the insertion of hydrated cations with different hydration shell structures. To stabilize the lamellar structure, hydrogel pillars that could form in the interlayer spacing during the cross-linking reaction between multivalent cations (including Ca^2+^, Ba^2+^, Mn^2+^, and Al^3+^ herein) and alginate molecules were incorporated in this study. As shown in Fig. 3c, d, the interlayer spacing of the Ca-SAT membrane is stably and effectively constrained at 16.2 ± 0.2 Å, and both structural expansion and contraction are suppressed. The effective diameter of the ion channel of the Ti_3_C_2_T_*x*_ membrane, which could be determined by subtracting the thickness of one rigid layer of Ti_3_C_2_T_*x*_ (8.8 Å), is considered to be 7.4 ± 0.2 Å^21^. Sequence analysis was also conducted to explore the effect of SA concentration and cross-linking cations on the structural stability (Fig. 3e). It could be concluded that when 1 wt% SA is added to the Ti_3_C_2_T_*x*_ nanoflakes, the coordination interaction between the SA molecules and cross-linking cations is unable to supply sufficient force to overcome the significant intercalation-induced structural changes. When the SA amount increased to 5%, the d-spacing fluctuation was distinctly suppressed to 0.52 Å; when the SA amount was further increased to 10%, the d-spacing remained almost constant in aqueous solution, which suggested that the membrane would have a more stable structure when more pillars were incorporated in the interlayer spacing. Considering that excess SA addition could influence the uniformity of the layer structure and obstruct the pathways, SA was set at 5–10 wt% in the nanosheet in the follow-up experiment. For the SA-Ti_3_C_2_T_*x*_ membrane cross-linked with other divalent cations (Ba^2+^ and Mn^2+^), the interlayer spacing could also be rigidly controlled (Fig. 3f). No apparent structural changes were observed after soaking in various solutions, and the effective diameters of both the Ba-SAT and Mn-SAT interchannels were fixed at 16.2 ± 0.1 Å, which is approximately the same as that of the Ca-SAT membrane. Different from the Ca-alginate gel with excellent flexibility, the Al-alginate gel was stiff but brittle (Supplementary Fig. 9), which could be attributed to the different cross-linking mechanisms^48^. Therefore, the incorporation of Al-alginate into the interlayer reduced the flexibility of the Ti_3_C_2_T_*x*_ membrane, and the Al-SAT membrane is not discussed in further detail because the brittle structural characteristics are not acceptable for industrial filtration processes.

### Ion-sieving behavior through the M-SAT membrane

**Fig. 4:**
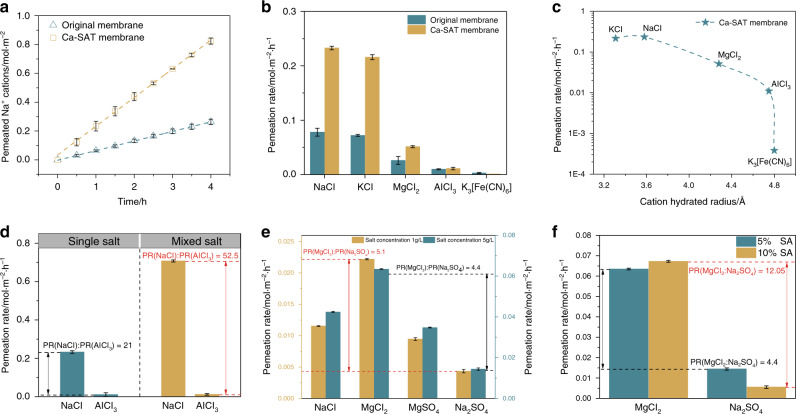
Ion permeation characteristics through original and pillared membrane membranes. **a** Number of Na^+^ ions permeated through the original and Ca-SAT membranes. **b** Permeation rate (PR) of the original and Ca-SAT membranes for different salts. **c** Relationship between PR and ion radius for the Ca-SAT membrane. **d** PR of 0.5 mol L^−1^ NaCl and AlCl_3_ single salt solution compared with that of mixed salt solution. **e** PR of the Mn-SAT membrane for different ion pairs (NaCl, MgCl_2_, MgSO_4_, and Na_2_SO_4_). **f** PR of the Mn-SAT membrane containing different amounts of SA for MgCl_2_ and Na_2_SO_4_. The error bars represent the standard deviations of three parallel tests.

The effect of stable interlayer spacing on the ion transport behavior through the Ti_3_C_2_T_*x*_ membrane pillared by Ca-alginate gel was first evaluated and compared with that of the pristine membrane. As shown in Fig. 4, the ion concentration increases approximately linearly with time under concentration-driven diffusion in both the Ca-SAT and original membranes, whereas the permeation rates (PRs, indicated by the slope of the curves) of cations with various valences exhibit a remarkable difference after the nanocapillaries are restricted. Both monovalent and divalent cations demonstrated an obvious acceleration when diffused through the pillared Ca-SAT membrane, and the increase in the PR varied with different valences. In particular, the monovalent cation permeability was enhanced by nearly three times that in the pristine Ti_3_C_2_T_*x*_ membrane and still maintained the highest PR. The PR of Al^3+^ through the Ca-SAT membranes was distinctly different from that of low-valence cations and increased only slightly with respect to the pristine membrane. The diffusion of Fe(CN)_6_^3−^ became dramatically slower after the Ca-alginate spacer was formed. We found that Fe(CN)_6_^3−^ can minimally permeate, even after 36 h of measurements. To further confirm the ion-sieving properties of the Ca-SAT membrane, a mixed solution of NaCl and AlCl_3_ with a concentration of 0.5 mol/L was filled into the feed compartment, and the PR difference was more remarkable than in separate diffusion experiments (Fig. 4d). The values of the Na^+^/Al^3+^ PR ratio for the Ca-SAT membrane were further increased from 21 to 52.5. We further studied the dependence of the cation PRs on the thickness of the Ca-SAT membrane (Supplementary Fig. 10). The ion PRs exhibited a distinct decrease with increasing thickness, which could be attributed to the prolonged permeation length. Nevertheless, we also observed that the ion-sieving property of the Ca-SAT membrane was dependent on the cation valence and was almost unaffected by the thickness variation. The concentration of the salt solution demonstrated no significant effect on the ion selectivity of the Ca-SAT membrane (Supplementary Fig. 11).

The effect of cross-linking cations on the ion selectivity of the membrane was determined by comparing the typical salt (NaCl and MgCl_2_) diffusion through membranes with different alginate hydrogel spacers (Supplementary Fig. 12). The SA-Ti_3_C_2_T_*x*_ membrane cross-linked with Ba^2+^ presents a cation permeability characteristic similar to that of the Ca-SAT membrane, whereas in the Mn-SAT membrane, the ion permeability no longer follows the above order. The NaCl permeability, which is consistent in the Ca- and Mn-SAT membranes, is minimally affected by cross-linking cations, whereas the MgCl_2_ permeability through the Mn-SAT membrane presents an obvious increase and becomes even slightly higher than that of Na^+^. For a comprehensive assessment of ion permeation through the Mn-SAT membrane, we conducted permeation experiments with different cation-anion pairs. As shown in Fig. 4e, for both Cl- and SO_4_^2−^, Mg^2+^ is observed to be more favorable to permeation through the membrane than Na^+^, while the PR difference is not significant. Moreover, the diffusion of chloride salt is faster than that of sulfate salt for either Na^+^ or Mg^2+^ cations in the Mn-SAT membrane, and the MgCl_2_/Na_2_SO_4_ selectivity of the Mn-SAT membrane reached 4.4. The effect of salt concentration on anion-determined transport behavior is also analyzed and clearly suggests that all ion permeabilities are enhanced by increasing the salt concentration. However, the MgCl_2_/Na_2_SO_4_ selectivity exhibits a steady decrease with increasing salt concentration, indicating that a lower concentration solution potentially contributes to the highly efficient anion sieving process. Furthermore, more SA molecules in the interlayer spacing is beneficial for improving the selectivity of MgCl_2_/Na_2_SO_4,_ which was further increased to 12.05 (Fig. 4f).

Structural stability is a critical parameter in practical ion-sieving applications; in particular, the laminate structure of the membrane was adjusted with angstrom-scale precision in this study. To provide a comprehensive evaluation of the stability of pillared Ti_3_C_2_T_*x*_ membranes, the as-prepared Ca-SAT membrane was subjected to a long-term immersion treatment. The membrane exhibited no apparent sign of disintegration or dissolution in deionized (DI) water even after 20 days (Supplementary Fig. 13a). The corresponding XRD results confirmed the consistent interlayer spacing and suggested that the stabilized structure was not affected by long-term immersion (Supplementary Fig. 13). It is also clear that the Na^+^ permeation rate through the Ca-SAT membrane was constant before and after the immersion treatment (Supplementary Fig. 13), implying that the ion-sieving performance would not deteriorate by long-term operation in an aqueous environment. Furthermore, during three permeation/drying cycles, ion permeation through the Ca-SAT membrane remained at a substantially constant rate (Supplementary Fig. 14). The XRD measurement results also certified that the stable lamellar structure could withstand the cyclic permeation/drying test and that the pillared Ti_3_C_2_T_*x*_ membrane demonstrated excellent structural durability. To further confirm the variation in the forms of Ca that existed and the concentration in the Ca-SAT membrane after ion permeation, XPS measurements were conducted. Consistent with the typical XPS spectrum of Ca-alginate gel in previous reports^49,50^, the Ca 2*p* region could be fitted by two peaks at binding energies of 350.6 and 347.0 eV, which suggested the cross-linking interaction between Ca^2+^ and alginate molecules (Supplementary Fig. 15). Moreover, the unchanged binding energies of Ca 2*p* peaks after ion permeation illustrated that no changes in Ca occurred, and the Ca-alginate pillars were firmly anchored in the interlayer spacing. In addition, according to the Ca depth profiles along the membrane thickness direction, the concentration of Ca contained in the membrane was basically unchanged before and after ion permeation, suggesting that the Ca-alginate pillar was not affected during the ion-sieving process (Supplementary Fig. 16). The constant Ca concentration with different depths also confirmed that the hydrogel pillars were homogeneously distributed in the membrane, consistent with the SEM result (Fig. 2f). The full XRD patterns of the Ca-SAT membrane after the permeation test confirmed that TiO_2_ anatase, which is the main oxidation product, was not formed during operation of the membrane (Supplementary Fig. 17)^51^. The high-resolution XPS spectra of Ti 2*p* also demonstrate that the proportion of TiO_2_ in the as-prepared Ca-SAT membrane was negligible, and the Ti form also remained constant, indicating that the membrane oxidation was slight^32^. The regeneration ability of the Ca-SAT membrane was also evaluated, as shown in Supplementary Fig. 18. Although permeation experiments with different cations were continuously performed on the same Ca-SAT membrane after the Na^+^ permeation test was first conducted, the permeation rate of Na^+^ could be effectively restored only by thorough rinsing using DI water, indicating excellent regenerability. Moreover, recent studies have also confirmed the outstanding antibacterial efficiency and chlorine tolerance of the Ti_3_C_2_T_*x*_ membrane^40,52^, which also provides evidence of the excellent regenerability of the Ti_3_C_2_T_*x*_-based membrane.

## Discussion

Extensive studies in nanofluidics have revealed that the size exclusion effect of hydrated ions in extremely confining environments cannot be generally considered in terms of a comparison of the ionic diameter with the transport channel size. Although theoretical studies have suggested that nanopores with diameters <10 Å should exhibit significant energy barriers because the hydrate shells around the ions could become deformed or might even be totally stripped so that the ions could enter and transport into the nanoconfined space^35,36^, Peng et al. recently obtained ultrahigh-resolution images of the structural changes of individual Na^+^ ions using a combined scanning tunneling microscopy and AFM system and proved that hydrated Na^+^ with five water molecules in the first hydration shell could exist in various metastable states with different hydration numbers and/or arrangement symmetries (standing or planar), which provides direct evidence for dehydrated transport in nanofluids^53^. As described above, the effective diameter of the nanochannel in the Ca-SAT membrane was fixed at 7.4 ± 0.2 Å, which is comparable with the monovalent alkali cations (for instance, Na^+^: 7.16 Å and K^+^: 6.62 Å) but smaller than most of the hydrated ions with a high valence (for instance, Mg^2+^: 8.56 Å and Al^3+^: 9.60 Å) (Supplementary Table 1), and the increasing ion concentration with time in the permeate compartment, even at a low feed concentration, could verify ion dehydration during diffusion through the pillared membrane.

**Fig. 5:**
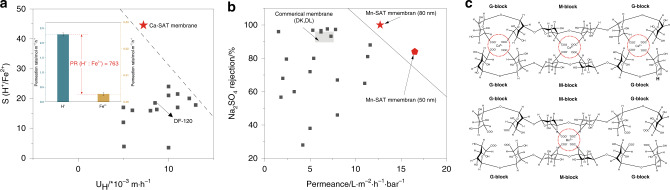
Ion-sieving performance comparison and SA cross-linking mechanism. **a** Comparison of the separation performance of the Ca-SAT membrane and previously reported ion-exchange membranes, as well as the commercial DF-120 membrane. For detailed experimental conditions, see Supplementary Table 2. **b** Comparison of the separation performance of the Mn-SAT membrane and previously reported nanofiltration membranes, as well as the commercial DK and DL membranes. For detailed experimental conditions, see Supplementary Table 3. **c** Schematic diagram of sodium alginate cross-linked with Ca^2+^ and Mn^2+^. The error bars represent the standard deviations of three parallel tests.

The energy barrier for ion dehydration is considered to be directly related to the magnitude of the hydration free energy, which depends on the electrostatic attraction between ions and polarized water molecules^12^. This suggests that the hydration free energy changes induced by ion valence are much more pronounced than those induced by ion radius, and the hydration energy of K^+^ (3.31 Å, −435 kJ mol^−1^) is only 100 kJ mol^−1^ higher than that of Na^+^ (3.58 Å, −333 kJ mol^−1^). However, for the multivalent cations Mg^2+^ (4.28 Å, −1868 kJ mol^−1^) and Al^3+^ (4.80 Å, −4665 kJ mol^−1^), these differences could amount to 1500 kJ mol^−1^ and 2700 kJ mol^−1^, respectively. As a result, when hydrated cations are ready to enter the stable nanochannel in the Ca-SAT membrane, alkali cations could experience a lower energy barrier and consequently exhibit a higher PR. However, Al^3+^, whose water shell is much ‘tougher’ than the shell of other ions, needed to overcome a significant energy barrier to diffuse through the atom-scale channel, thereby exhibiting a significantly slower diffusion rate than that of the monovalent cations. In addition, it is noticeable that although the channel diameter difference between the Ca-SAT membrane and Na-intercalated Ti_3_C_2_T_*x*_ membrane is only ~2 Å, which is merely 1/5 of the hydrated Na^+^ diameter, the PR shows a sharp increase, which indicates the importance of channel size when it is reduced to the nanometer scale, consistent with the experimental observation by Geim and coworkers in which the hydrated ion PR might follow an exponential dependence on the channel radius under nanoscale confinement^12^. Based on the favorable cation sieving capability shown above, further investigation on the application of the Ca-pillared Ti_3_C_2_T_*x*_ membrane in the recovery of acids from iron-based wastewater was performed. The results revealed that the PR of H^+^ in the Ca-SAT membranes with a thickness of 5 μm is ~800 orders of magnitude larger than that of Fe^2+^ using typical industrial wastewater and Ca-SAT membranes without any further treatment, and the permeation process exhibited a performance superior to the traditional diffusion dialysis processes conducted by polymer-based ion-exchange membranes, including commercial DF-120 membranes (Fig. 5a and Supplementary Table 2). In particular, the notably satisfactory separation factor effectively avoids Fe leakage, which is considered a main limitation of polymeric anion-exchange membranes. We believe this characteristic can be significantly improved by decreasing the membrane thickness or by other surface modifications.

The diameter of the nanochannel in the Mn-SAT membrane is comparable with that of the Ca-SAT membrane, whereas the ions exhibit completely different transport characteristics. Recent research revealed that the cross-linking processes of SA molecules with different divalent cations are not identical because the alginate affinity varies with cation type^54^. Hence, Ba^2+^ and Ca^2+^ cations could bind with both G-blocks and M-blocks in the SA molecule through coordination bonds to form a stable chelate, whereas stable coordination occurs only between Mn^2+^ and G-blocks, and the binding of Mn^2+^ and M-blocks, which is primarily based on electrostatic attraction, is relatively labile (Fig. 5c). As a result, the carboxyl group in M-blocks could remain on the composite SA-Ti_3_C_2_T_*x*_ nanosheet, leading to a more negatively charged surface of the Mn-SAT capillaries, as confirmed by the XPS and zeta potential results. However, we also noticed that compared with polymeric membranes with zeta surface potentials typically ranging from −40 to −60 mV^55^, the electronegativity of the Mn-SAT membrane surface was not as obvious (−3.7 mV at pH 7). Furthermore, only a 4-mV surface potential difference between the Mn- and Ca-SAT membranes led to a completely opposite permeability sequence of Na^+^ and Mg^2+^. Instead of applying the traditional models proposed for polymeric membranes, a comprehensive analysis is conducted in the following section to illustrate how the small differences in surface potentials of angstrom-scale channel walls affect the intermediate ion distribution and interaction, thus affecting its contribution to the different ion-sieving performance of the Mn-SAT membrane.

According to Gouy–Chapman theory, the potential distribution in the interfacial electrical double layer (EDL) is characterized by the surface potential (*ψ*_*surf*_) of the charged surface, the potential drop (*ψ*_*drop*_) over the Stern layer and the zeta potential (ζ) at the shear plane, which can be expressed as equation (1):

1 $$\psi _{{\mathrm{sur}}f}\,=\,\psi _{drop}\,+\,\zeta.$$

From molecular dynamics simulation results, the concentration of co-ions is negligible in the Stern layer compared with the concentration of counterions, and the counterions are generally immobilized due to strong Coulomb forces^56,57^. Because the thickness of the diffusion layer (Debye length) is much greater than that of the Stern layer, the Debye length deduced from Poisson–Boltzmann theory is normally simply treated as the EDL thickness in micro- and nanoscale channels, and the overlap of Debye layers could significantly affect the ion transport process^58^. Nevertheless, because the effective diameter of the Mn-SAT channel is notably confined to the angstrom scale, together with the existence of the first water layer that could hinder hydrated cations from moving towards the charged surface, the Stern layer (the outer Helmholtz layer (OHL), in this study) occupies a nonnegligible space in this channel, which could also have a profound impact on the intermediate ion transport behavior. In negatively charged Mn-SAT capillaries, the *ψ*_*drop*_ across the OHL is essentially determined by the electric charge of the counterions adsorbed next to the first water layer. Compared with the monovalent cation Na^+^, the negatively charged surface with attached bivalent cations Mg^2+^ is expected to have a relatively large *ψ*_*drop*_ at the Stern layer, subsequently generating a small *z*-value at the shear plane. Consequently, under this partially screened electrical field, the mobile Mg^2+^ in the Debye layer could be less sensitive to the charged surface and permeate more rapidly through the membrane along the concentration gradient. Simultaneously, the relatively low ionic density of Mg^2+^ is necessary to neutralize the same amount of surface charge, which also leads to a thinner Stern layer and, consequently, a wider transport channel that also contributes to the higher Mg^2+^ PR. Despite the high energy barrier, the surface charge mechanism dominated the ion transport under severe confinement and caused the difference between the Mg^2+^/Na^+^ PRs in the Mn-SAT membrane. Interestingly, we noted that the NaCl PRs were nearly unchanged through the Ca- and Mn-SAT membranes due to the balance between the electrostatic repulsion and the attraction of co/counterions to the membrane surface. For chloride salts and sulfate salts with the same cations, due to the stronger electrostatic repulsion of SO_4_^2−^ co-ions than Cl^−^ against the negatively charged channel wall when transported through the Mn-SAT membrane, divalent SO_4_^2−^ diffused slower than monovalent Cl^−^. For the electroneutrality constraint (Supplementary Fig. 19), the cations (Na^+^) and anions would both intercalate and diffuse through the membrane in a stoichiometric ratio, and NaCl exhibited a higher PR than Na_2_SO_4._ To further confirm the roles of channel wall surface charges, we evaluated the Na_2_SO_4_/water selectivity in pressure-driven filtration measurements with different membrane thicknesses. As shown in Fig. 5b and Supplementary Table 3, the Na_2_SO_4_ rejection rate of the Mn-SAT membrane with a thickness of only 50 nm could reach 84%, and as the membrane thickness further increased to 80 nm, Na_2_SO_4_ could hardly permeate through the pillared membrane. Due to the structural characteristics of the top functional layer formed during interfacial polymerization, ion sieving of the traditional polymeric nanofiltration membrane relies principally on the surface charge density. However, for the lamellar membrane, under the surface electrostatic effect, the ion transport behavior under extreme nanoconfinement would be significantly and efficiently affected by the channel wall potential, and the ultrathin Mn-SAT membrane could achieve an outstanding Na_2_SO_4_ rejection rate and overcome the salt permeability and water/salt selectivity trade-off observed for state-of-the-art nanofiltration membranes.

This work confirmed the effectiveness and convenience of this approach to stabilize the interlayer spacing of the lamellar architecture at the angstrom scale, which was attributed to the abundant surface functional groups and cross-linking property of SA molecules. This facile strategy could be easily extended because comparable polymers including chitosan, gelatin, and methylcellulose and other MXene materials could be used to construct stable nanochannels with different surface and structural characteristics; in particular, more than 30 MXene compositions have been published as the 2D MXene family grows^20,22^. Accordingly, different structural and chemical properties would contribute insights into the mechanism of ion transport under extreme confinement. Furthermore, as mentioned above, the ion transport behavior could be significantly affected by the surface properties of the lamellar membranes^43,45^, and all the Ti_3_C_2_T_*x*_ membranes pillared by different hydrogels reported herein could act as favorable substrates for the construction of asymmetric membranes, with the ion-sieving performance further improved by advanced surface modification.

In summary, we demonstrated a strategy for restricting the structural changes of lamellar MXene membranes and obtained a significantly enhanced ion-sieving property. The different alginate hydrogel pillars formed between the adjacent nanosheets effectively stabilized the size of the interlayer spacing at ~7.4 ± 0.2 Å, but the ion permeation exhibited distinct differences depending on the multivalent cations cross-linked with alginate molecules. Attributed to the enhanced permeability of monovalent cations without simultaneous loss of the blocking effect to high-valence cations, the Ca-alginate pillared Ti_3_C_2_T_*x*_ membrane presents a clear permeation cutoff and notable selectivity dependence on the valence of cations, which makes the Ca-SAT membrane a promising candidate in the steel industry for recovery of acids from iron-containing wastewater. Additional systematic studies could be conducted to explore other applications and to compete with state-of-the-art polymer-based ion-exchange membranes or nanofiltration membranes. Moreover, the Mn-alginate pillared membrane shows a different ion-sieving property towards anion valence, which is attributed to the arrangement and interaction of crowded counterions under extreme confinement. The ultrathin Mn-alginate membrane with an 80-nm thickness demonstrated a 100% rejection rate of Na_2_SO_4_ with high water permeance, whose performance was far beyond the limit of state-of-the-art nanofiltration membranes. Based on the significant effect of the surface property of the severely confined channel on the ion transport behavior confirmed here, our work introduces a novel and efficient approach to improve ion selectivity, which might be of interest in many promising applications in energy- and environment-related fields.

## Methods

### Preparation of Ti3C2Tx nanosheets

Ti_3_C_2_T_*x*_ nanosheets were synthesized according to the minimally intensive layer delamination method reported previously^20,59^. The etchant solution was prepared by completely dissolving 1 g LiF in 20 ml of 9 mol L^−1^ hydrochloric acid in a 50 ml plastic vial. Subsequently, 1 g Ti_3_AlC_2_ powder was gradually added into the etchant solution, and the mixture was stirred at 500 rpm at 45 °C for 24 h. The acidic product was rinsed with DI water via centrifugation (5 min per cycle at 3500 rpm) until the pH of the supernatant reached a value of 5. The clay-like sediment was redispersed into DI water and subjected to vigorous manual shaking to delaminate the Ti_3_C_2_T_*x*_ multilayers (Supplementary Fig. 20). After centrifugation at 4000 rpm for 60 min, the supernatant containing monolayer Ti_3_C_2_T_*x*_ nanosheets was collected.

### Fabrication of the M-SAT membrane

SA solution (0.01 mg mL^−1^) was mixed with diluted Ti_3_C_2_T_*x*_ colloidal solution (0.01 mg/ml) and stirred for 12 h at room temperature (RT), and composite SA-Ti_3_C_2_T_*x*_ nanosheets were assembled into the hybrid SA-Ti_3_C_2_T_*x*_ membrane with a lamellar structure using the vacuum-assisted filtration (VAF) method through a polyvinylidene fluoride (PVDF) membrane (pore size of 0.22 μm)^28^. The SA-Ti_3_C_2_T_*x*_ membrane was immersed into different multivalent M^n+^ cross-linking solutions (Ca^2+^, Ba^2+^, Mn^2+^, and Al^3+^, 0.1 mol L^−1^) for 4 h to obtain the cross-linked membrane with hydrogel pillars in the interlayer spacing. After drying at RT under vacuum, the flexible membrane was peeled from the PVDF supporter and was stored under vacuum for further testing. To eliminate the impact of structural defects during the nanosheet stacking process, which is critical for a highly efficient separation process, the thickness of all membranes used in the current experiments was fixed at 5 μm without special instructions.

### Interlayer spacing measurement

The original and pillared Ti_3_C_2_T_*x*_ membranes were immersed in pure water and different aqueous chloride solutions (Li^+^, Na^+^, K^+^, Rb^+^, Mg^2+^, Ca^2+^, Al^3+^, and NH_4_^+^, 0.5 mol/L) for 1 h, ensuring that a sufficient number of ions intercalated into the interspacing. XRD measurements were conducted immediately after gently wiping the free water from the membrane surface^12,40^.

### Ion permeation measurement

Ion permeation experiments were performed with a homemade U-shaped permeability apparatus that was divided into feed and permeate compartments (Supplementary Fig. 21). The feed half of the cell was filled with different salt solutions, and the permeate half contained the same volume of DI water. A peristaltic pump was selected to exchange the solution around the membrane surface with the bulk solution to avoid the concentration polarization effect. The flow rate of the pump was set to 24 mL min^−1^, which allowed circulation of the entire solution in each compartment in 5 min. To guarantee the accuracy of the results, parallel experiments were performed three times under each set of conditions, and permeation tests of the feed compartment with 0.5 mol L^−1^ NaCl were used as a standard and performed before and after every ion permeation measurement. The conductivity of the permeate side was measured by a conductivity meter over time to calculate the ion concentration, which showed good agreement with the concentration value derived from inductively coupled plasma (ICP) mass spectrometry (Supplementary Fig. 22). To compare the ion-sieving property of the Ca-SAT membrane with other membranes, for different chloride salts, the feed concentrations were 0.5 mol L^−1^, and for K_3_Fe(CN)_6_, the concentration was 5 g L^−1^ ^13^. To be consistent with the commercial nanofiltration membrane evaluation method, salt concentrations of 1, 5, and 15 g L^−1^ were tested on Mn-SAT membranes^32^.

### Diffusion dialysis of the HCl/FeCl_2_ solution system

A typical wastewater sample composed of a mixed aqueous solution of 1 mol L^−1^ HCl and 0.1 mol L^−1^ FeCl_2_ was used in the diffusion dialysis tests for acid recovery. The concentration of HCl was determined by titration with a standard Na_2_CO_3_ solution using methyl orange as an indicator, while the concentration of FeCl_2_ was directly determined by ICP. To compare with traditional polymer-based ion exchange, the separation factor (S) with respect to one species over another is given as the ratio of dialysis coefficients (U) of the two species present in the solution.$U_{H^ + }$and$U_{F{\mathrm{e}}^{2 + }}$ were calculated by the following Eq. (2)^60,61^:

2 $$U\,=\,\frac{M}{{A{\mathrm{t}}\Delta C}},$$

where *M* is the amount of substance transported in moles, *A* is the effective area in square meters, *t* is the time in hours, and ΔC is the logarithmic average concentration between the two chambers in moles per cubic meter and defined as Eq. (3):

3 $$\Delta C\,=\,\frac{{{C_{\rm{f}}^{\rm{o}}}\,-\,\left( {{C_{\rm{f}}^{\rm{t}}}\,-\,{C_{\rm{d}}^{\rm{t}}}} \right)}}{{\ln \left[ {\frac{{C_{\rm{f}}^{\rm{o}}}}{{C_{\rm{f}}^{\rm{t}}\,-\,C_{\rm{d}}^{\rm{t}}}}} \right]}}.$$

The separation factor (*S*) of $U_{H^ + }$and $U_{F{\mathrm{e}}^{2 + }}$ was obtained by taking the ratio of dialysis coefficients according to the following Eq. (4):

4 $$S\,=\,\frac{{U_{H^ + }}}{{U_{F{\mathrm{e}}^{{\mathrm{2}} + }}}}.$$

### Pressure-driven filtration measurement

The Mn-SAT membrane water/Na_2_SO_4_ separation performance was tested by homemade nanofiltration equipment working in dead-end filtration mode (Supplementary Fig. 23). The Mn-SAT membrane with thicknesses of 50 and 80 nm was fabricated by the VAF method through a mixed cellulose membrane (pore size of 0.22 μm). The effective filtration area was 0.785 cm^2^ (a circular hole with a radius of 0.5 cm), and all experiments were performed at RT. The concentration of salt solution (Na_2_SO_4_) was 50 ppm, and the pressure of the experiment was fixed at 1 bar with controllably pressurized compressed air. We collected the permeated fluid after 1 h of the experiment for stabilization. The water permeation and salt rejection are calculated as follows Eq. (5) and (6):

5 $${\mathrm{Permeance}}\,=\,\frac{V}{{{A}\,\times\,{t}\,\times\,{P}}},$$

6 $${R}\,=\,\left( {1\,-\,\frac{{{C}_{\mathrm{p}}}}{{{C}_{\mathrm{f}}}}} \right)\,\times\,100{\mathrm{\% }},$$

where *V* is the volume of permeate collected (L), *A* is the membrane effective area (m^2^), *t* is the permeation time (h), *P* is the applied pressure (bar), and *C*_p_ and *C*_f_ are the concentrations of the permeate and feed solutions, respectively.

### Material characterization

The Ti_3_AlC_2_ powders, Ti_3_C_2_T_*x*_ nanosheets and d-spacing values were characterized by XRD (Ultima lV, Japan) with Cu Kα radiation at a step of 0.02° and a collection time of 6 s/step. SEM (Zeiss Gemini SEM 300, Germany) was used to study the surface topography and structural characteristics of the nanosheets and membranes. AFM (Bruker Multimode 8) was used to obtain a topographical image of the nanosheets in tapping mode. The morphology of the nanosheets was characterized by TEM (JEOL JEM-F200, Japan). XPS analysis was performed using a Thermo Fisher ESCALAB Xi^+^ instrument with monochromated Al-Kα radiation. FTIR characterization was performed using a Bruker VERTEX 33 unit over the wavenumber range of 1500–4000 cm^−1^. In situ infrared absorption spectroscopy measurements were conducted in transmission geometry by using a Thermo Scientific Nicolet iN10. The solid surface zeta potential was measured by a solid surface analyzer using Anton Paar SurPASSTM 3, and the colloidal solution zeta potential was measured by a laser particle size analyzer using a Zetasizer Nano ZS 90.

## Supplementary information

Supplementary Information

Peer Review File

## Data Availability

The data that support the findings of this study are available from the corresponding author upon reasonable request. Source data are provided with this paper.

## Source data

Source Data

## References

[1] Nair RR, Wu H, Jayaram PN, Grigorieva IV, Geim AK (2012). Unimpeded permeation of water through helium-leak-tight graphene-based membranes. Science.

[2] Joshi R (2014). Precise and ultrafast molecular sieving through graphene oxide membranes. Science.

[3] Cheng C, Jiang G, Simon GP, Liu Z, Li D (2018). Low-voltage electrostatic modulation of ion diffusion through layered graphene-based nanoporous membranes. Nat. Nanotechnol..

[4] Mouterde T (2019). Molecular streaming and its voltage control in Ångström-Scale channels. Nature.

[5] Koenig SP, Wang L, Pellegrino J, Bunch JS (2012). Selective molecular sieving through porous graphene. Nat. Nanotechnol..

[6] Surwade S (2015). Water desalination using nanoporous single-layer graphene. Nat. Nanotechnol..

[7] Zhao J (2019). Etching gas-sieving nanopores in single-layer graphene with an angstrom precision for high-performance gasmixture separation. Sci. Adv..

[8] Huang S (2018). Single-Layer graphene membranes by crack-free transfer for gas mixture separation. Nat. Commun..

[9] Raidongia K, Huang J (2012). Nanofluidic ion transport through reconstructed layered materials. J. Am. Chem. Soc..

[10] Kim H (2013). Selective gas transport through few-layered graphene and graphene oxide membranes. Science.

[11] Liu G, Jin W, Xu N (2016). Two-dimensional-material membranes: a new family of high-performance separation membranes. Angew. Chem..

[12] Abraham J (2017). Tunable sieving of ions using graphene oxide membranes. Nat. Nanotechnol..

[13] Yang Q (2017). Ultrathin graphene-based membrane with precise molecular sieving and ultrafast solvent permeation. Nat. Mater..

[14] Abozar A, Phillip S, Samuel T, Martin (2016). Large-area graphene-based nanofiltration membranes by shear alignment of discotic nematic liquid crystals of graphene oxide. Nat. Commun..

[15] Sun P (2013). Selective ion penetration of graphene oxide membranes. Acs Nano.

[16] Liu Y, Wang N, Cao Z, Jürgen C (2014). Molecular sieving through interlayer galleries. Mater. Chem..

[17] Deng M, Kwac K, Li M, Jung Y, Park HG (2017). Stability molecular sieving, and ion diffusion selectivity of a lamellar membrane from 2D molybdenum disulfide. Nano Lett..

[18] Sun L, Huang H, Peng X (2013). Laminar MoS_2_ membranes for molecule separation. Chem. Commun..

[19] Chen C (2018). Functionalized boron nitride membranes with ultrafast solvent transport performance for molecular separation. Nat. Commun..

[20] Yury G, Babak A (2019). The rise of MXenes. Acs Nano.

[21] Anasori B, Lukatskaya MR, Gogotsi Y (2017). 2D metal carbides and nitrides (MXenes) for energy storage. Nat. Rev. Mater..

[22] Anasori B (2015). Two-dimensional, ordered, double transition metals carbides (MXenes). ACS Nano.

[23] Lao J, Lv R, Gao J, Wang P (2018). Aqueous stable Ti_3_C_2_ MXene membrane with fast and photo-switchable nanofluidic transport. ACS Nano.

[24] Zheng S, Tu Q, Urban JJ, Li S, Mi B (2017). Swelling of graphene oxide membranes in aqueous solution: characterization of interlayer spacing and insight into water transport mechanisms. ACS Nano.

[25] Frey NC (2019). Prediction of synthesis of 2D metal carbides and nitrides (MXenes) and their precursors with positive and unlabeled machine learning. ACS Nano.

[26] Sarycheva A (2018). 2D titanium carbide (MXene) for wireless communication. Sci. Adv..

[27] Mendoza-Sánchez B, Gogotsi Y (2016). Synthesis of two-dimensional materials for capacitive energy storage. Adv. Mater..

[28] Shahzad F (2016). Electromagnetic interference shielding with 2D transition metal carbides (MXenes). Science.

[29] Liu H (2015). A novel nitrite biosensor based on the direct electrochemistry of hemoglobin immobilized on MXene-Ti_3_C_2_. Sens. Actuators B Chem..

[30] Ding. L (2017). Two-dimensional lamellar membrane: MXene nanosheet stacks angew. Chem. Int. Ed..

[31] Ren C (2015). Charge- and size-selective ion sieving through Ti_3_C_2_T_x_ MXene membranes. Phys. Chem. Lett..

[32] Lu S (2019). Self-crosslinked MXene (Ti_3_C_2_T_*x*_) membranes with good antiswelling property for monovalent metal ion exclusion. ACS Nano.

[33] Cohen-Tanugi D, McGovern RK, Dave SH, Lienhard JH, Grossman JC (2014). Quantifying the potential of ultra-permeable membranes for water desalination. Energy Environ. Sci..

[34] Jain T (2015). Heterogeneous sub-continuum ionic transport in statistically isolated graphene nanopores. Nat. Nanotech.

[35] Thomas M, Corry B, Hilder TA (2014). What have we learnt about the mechanisms of rapid water transport, ion rejection and selectivity in nanopores from molecular simulation. Small.

[36] Richards LA, Schafer AI, Richards BS, Corry B (2012). The importance of dehydration in determining ion transport in narrow pores. Small.

[37] Mashtalir O (2013). Intercalation and delamination of layered carbides and carbonitrides. Nat. Commun..

[38] Ghidiu M, Lukatskaya MR, Zhao M, Gogotsi Y, Barsoum MW (2014). Conductive two-dimensional titanium carbide ‘Clay’ with high volumetric capacitance. Nature.

[39] Chen L (2017). Ion sieving in graphene oxide membranes via cationic control of interlayer spacing. Nature.

[40] Ding L (2020). Effective ion sieving with Ti_3_C_2_T_*x*_ MXene membranes for production of drinking water from seawater. Nat. Sustain.

[41] Thebo KH (2018). Highly stable graphene-oxide-based membranes with superior permeability. Nat. Commun..

[42] Hung W (2014). Cross-linking with diamine monomers to prepare composite graphene oxide-framework membranes with varying D-Spacing. Chem. Mater..

[43] Hu M, Mi B (2013). Enabling graphene oxide nanosheets as water separation membranes. Environ. Sci. Technol..

[44] Zhang Y, Zhang S, Chung T (2015). Nanometric graphene oxide framework membranes with enhanced heavy metal removal via nanofiltration. Environ. Sci. Technol..

[45] Halim J (2016). X-ray photoelectron spectroscopy of select multi-layered transition metal carbides (MXenes). Appl. Surf. Sci..

[46] Zhang M (2019). Controllable ion transport by surface-charged graphene oxide membrane. Nat. Commun..

[47] Levi MD (2015). Solving the capacitive paradox of 2D MXene using electrochemical quartz-crystal admittance and in situ electronic conductance measurements. Adv. Energy Mater..

[48] Brus J (2017). Structure and dynamics of alginate gels cross-linked by polyvalent ions probed via solid state NMR spectroscopy. Biomacromolecules.

[49] Zhang MJ (2018). Mechanistic insights into alginate fouling caused by calcium ions based on terahertz time-domain spectra analyses and DFT calculations. Water Res..

[50] Guo ZW (2020). Fabrication of efficient alginate composite beads embedded with N-doped carbon dots and their application for enhanced rare earth elements adsorption from aqueous solutions. J. Colloid Interface Sci..

[51] Li ZT (2015). Synthesis and thermal stability of two-dimensional carbide MXene Ti_3_C_2_. Mater. Sci. Eng. B.

[52] Rasool K (2017). Efficient antibacterial membrane based on two-dimensional Ti_3_C_2_T_*x*_ (MXene) nanosheets. Sci. Rep..

[53] Peng J (2018). The effect of hydration number on the interfacial transport of sodium ions. Nature.

[54] Agulhon P, Markova V, Robitzer M, Françoise Q, Tzonka M (2012). Structure of alginate gels: interaction of diuronate units with divalent cations from density functional calculations. Biomacromolecules.

[55] Boya X. *The development of carboxylic acid separation by nanofiltration membrane for carboxylate platform using lingnocellulosic biomass*. The Pennsylvania State University 53–55 (2014).

[56] Wu J, Gerstandt K, Majumder M, Zhan X, Hinds BJ (2011). Highly efficient electroosmotic flow through functionalized carbon nanotube membranes. Nanoscale.

[57] Li J, Peng R, Li DQ (2019). Effects of ion size, ion valence and pH of electrolyte solutions on EOF velocity in single nanochannels. Anal. Chim. Acta.

[58] Bocquet Lydéric, Charlaix E (2010). Nanofluidics from bulk to interfaces. Chem. Soc. Rev..

[59] Alhabeb M (2017). Guidelines for synthesis and processing of 2D titanium carbide (Ti_3_C_2_T_*x*_ MXene). Chem. Mater..

[60] Liu X (2016). Porous diffusion dialysis membranes for rapid acid recovery. J. Mater. Sci..

[61] Ji W (2020). Self-organized nanostructured anion exchange membranes for acid recovery. Chem. Eng. J..

