# Supplementary material for: Ion sieving by a two-dimensional Ti3C2Tx alginate lamellar membrane with stable interlayer spacing
Source: Nat Commun. 2020 Jul 15;11:3540. doi: 10.1038/s41467-020-17373-4 (PMC7363915; doi:10.1038/s41467-020-17373-4)
Supplement: Supplementary file 1 — Supplementary Information [file 41467_2020_17373_MOESM1_ESM.pdf]

Supplementary Information

for

Ion Sieving by a Two Dimensional $Ti_3C_2T_x$ Alginate Lamellar Membrane with Stable Interlayer Spacing

Wang *et al.*

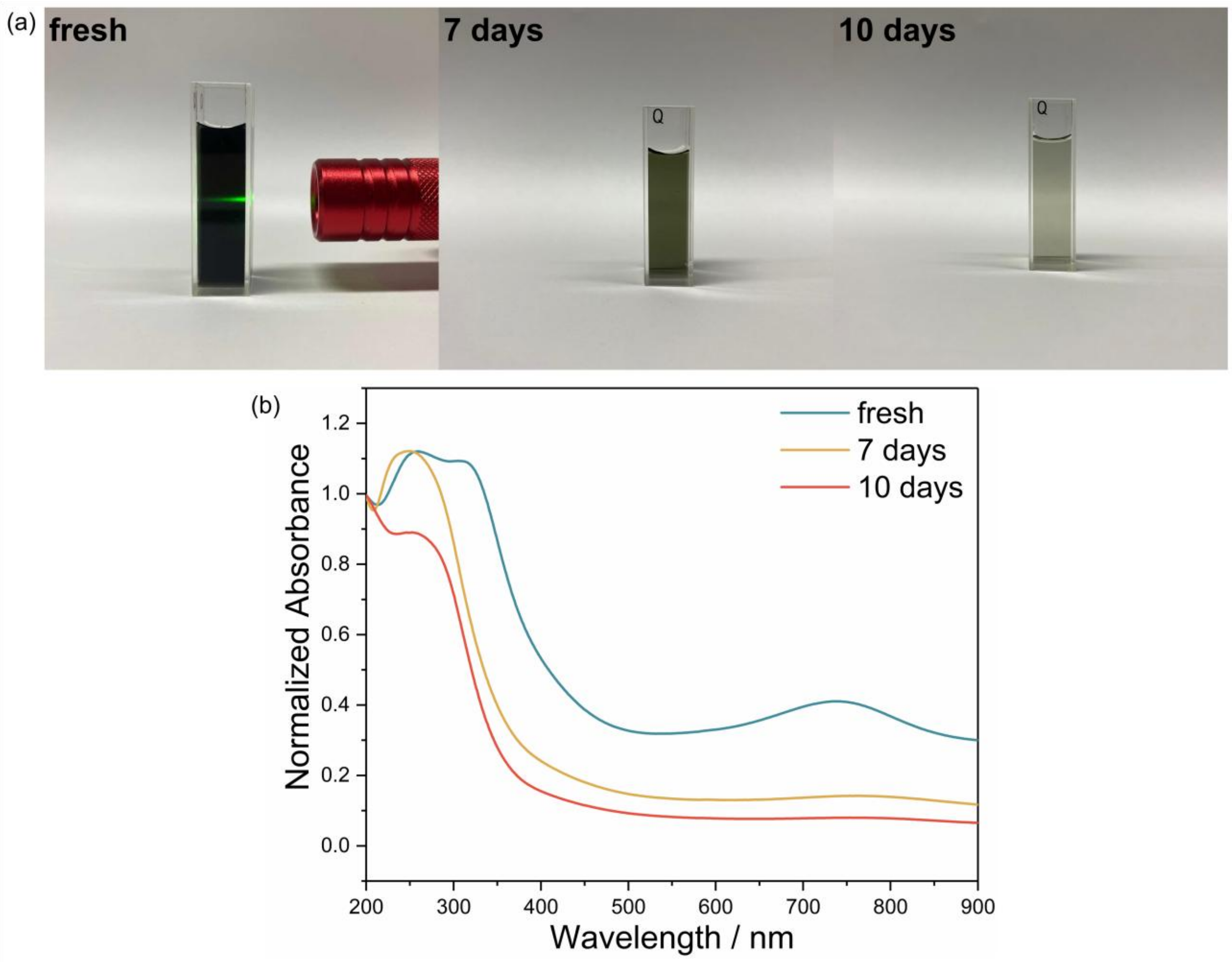

Supplementary Figure 1. Stability of the $Ti_3C_2T_x$ nanosheet dispersion stored at RT (a) Optical images of the dispersion changes over time (b) Normalized UV-vis spectra of the $Ti_3C_2T_x$ dispersion with different aging times. We observed that the color of the $Ti_3C_2T_x$ solution changed from dark green to transparent as it underwent degradation. Correspondingly, in the UV-vis spectra, the intensity of the absorption peak at 750 nm, which was usually chosen as the metric for the concentration of the $Ti_3C_2T_x$ nanosheets, exhibited a distinct decrease over time, and the broad absorption bump within the range of 220-325 nm also showed a clear shift to the left and became sharper over time[1-2].

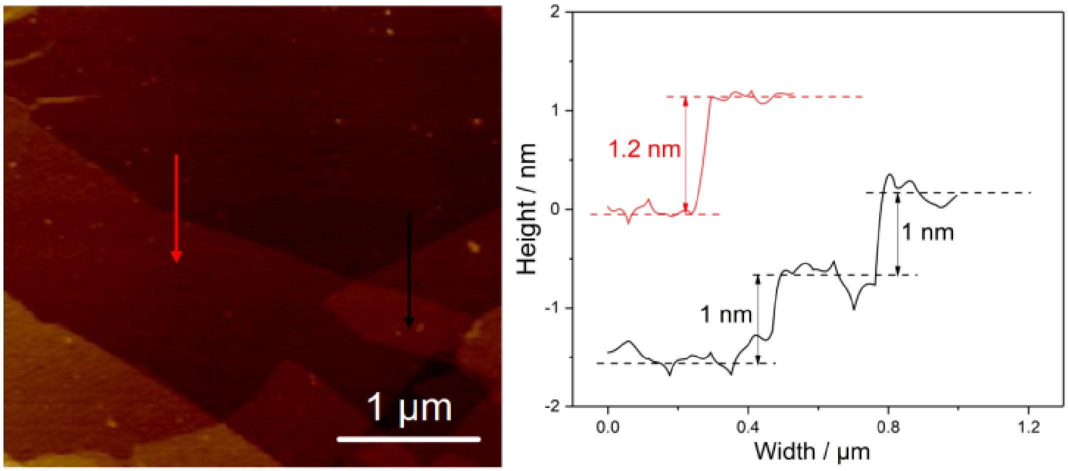

Supplementary Figure 2. AFM image of the $Ti_3C_2T_x$ nanosheets and the height profile of the nanosheets corresponding to the AFM image.

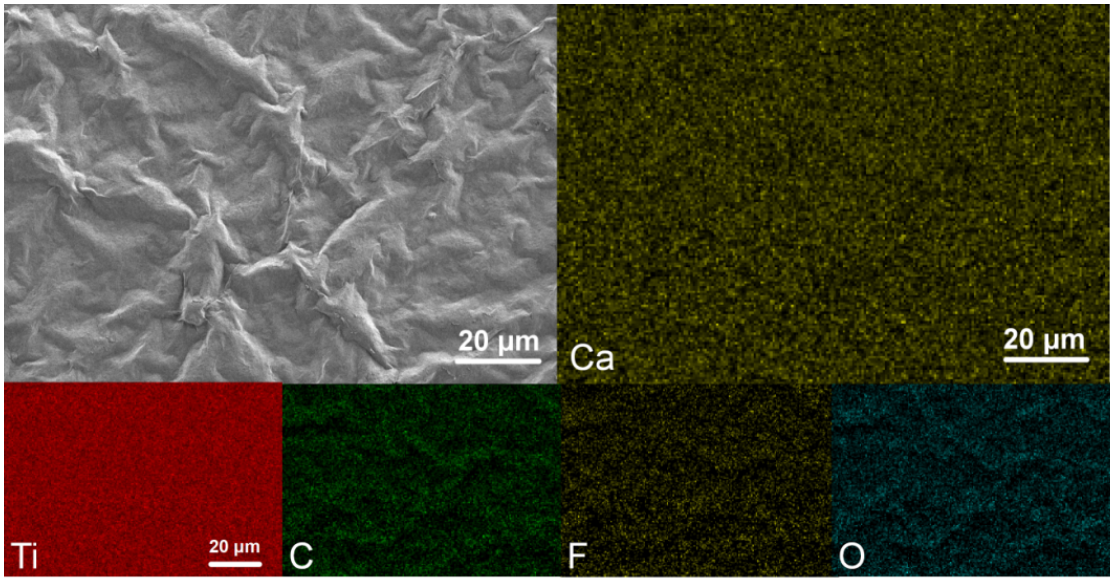

Supplementary Figure 3. SEM image of the surface of the Ca-SAT membrane and elemental maps of the same Ca-SAT membrane.

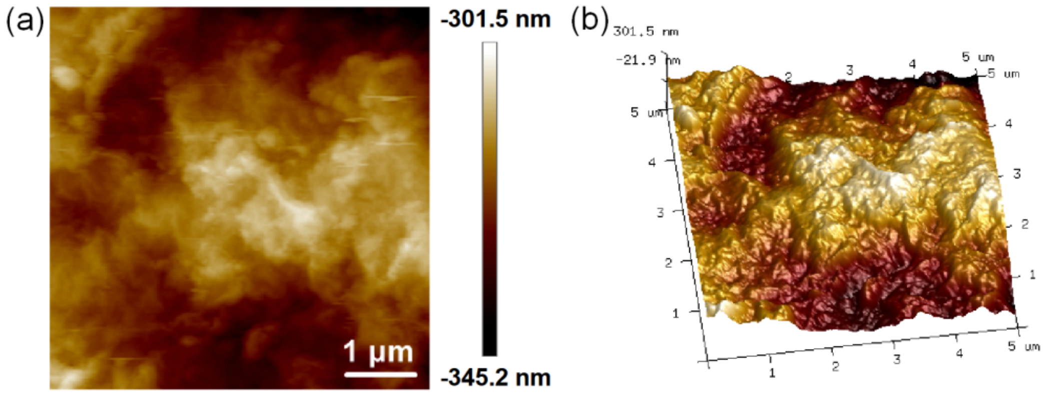

Supplementary Figure 4. AFM images of the pillared $Ti_3C_2T_x$ membrane surface (a) AFM images of the Ca-SAT membrane surface with a scan area of 5 μm × 5 μm. (b) The 3D height image corresponding to the same scan area.

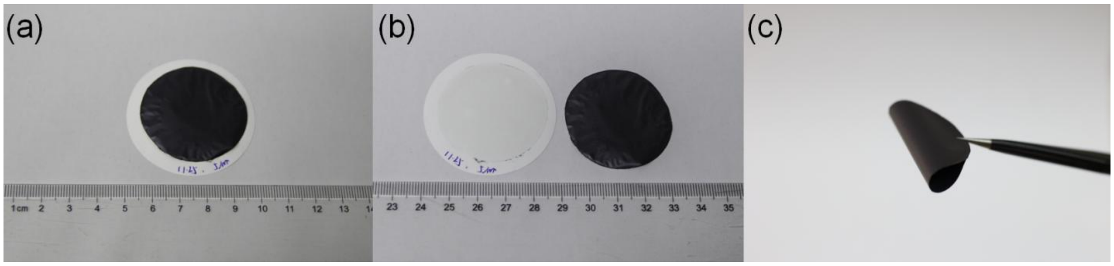

Supplementary Figure 5. Free-standing and flexible Ca-SAT membrane after peeling from the PVDF substrate.

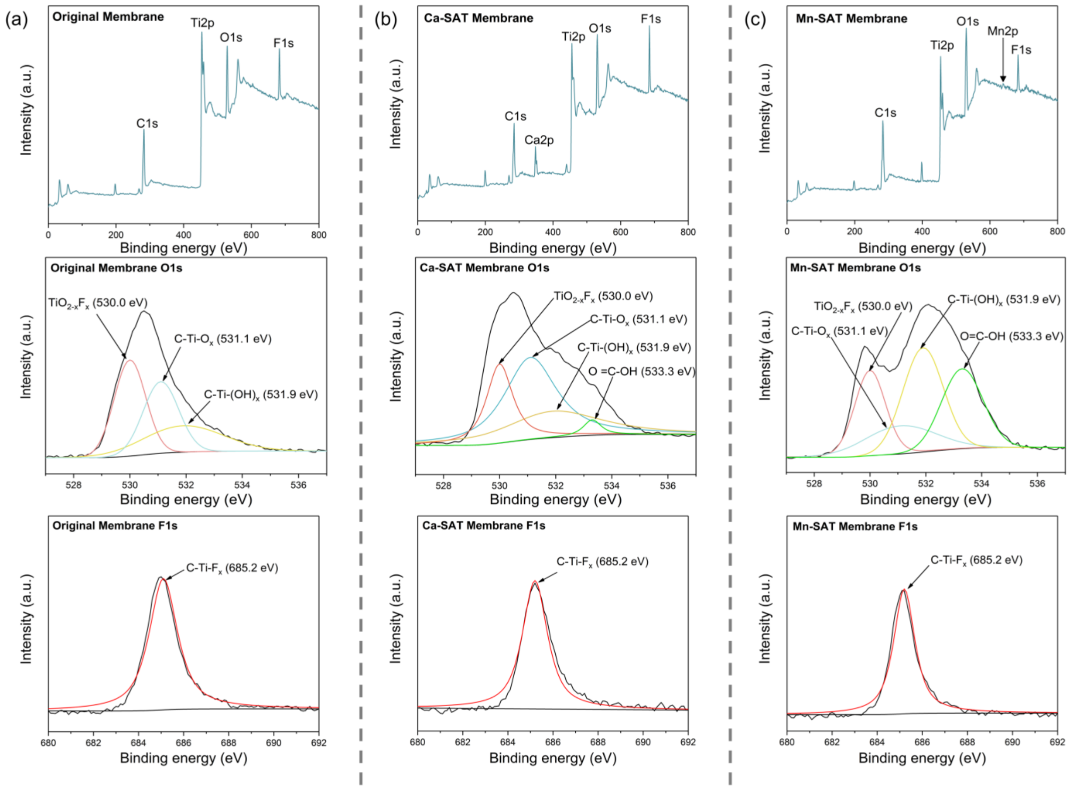

Supplementary Figure 6. The XPS analysis presents the chemical states of the elements on the surface of the original Ca-SAT and Mn-SAT membranes. XPS results, O 1s, ~~Ti 2p~~ and F 1s of the (a) original, (b) Ca-SAT and (c) Mn-SAT membranes.

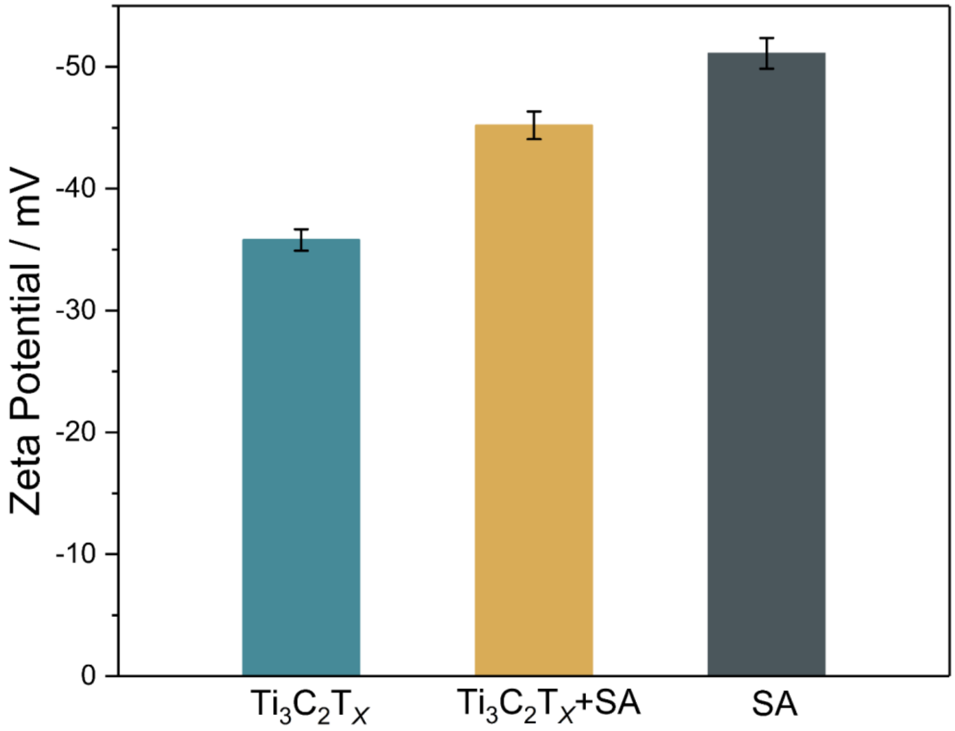

Supplementary Figure 7. Zeta potentials of the $Ti_3C_2T_x$ nanosheet solution, SA solution and mixed solution. The zeta potentials of the $Ti_3C_2T_x$ nanosheet colloidal solution (0.1 mg/ml) and SA solution (1 mg/ml) were -35 mV and -52 mV, respectively, and the zeta potential of the SA-$Ti_3C_2T_x$ mixed solution changed to -45 mV, which indicated that hydrogen bonds formed between the oxygen-containing surface terminal groups. The error bars represent the standard deviations.

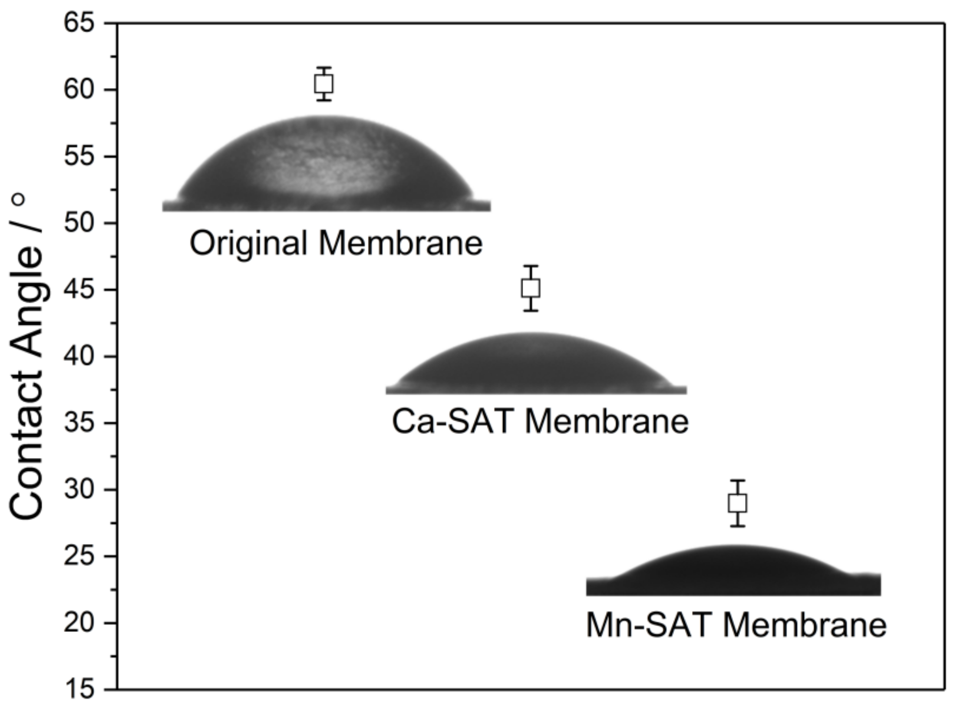

Supplementary Figure 8. Contact angle of water on the original, Ca-SAT and Mn-SAT membrane surfaces. The error bars represent the standard deviations.

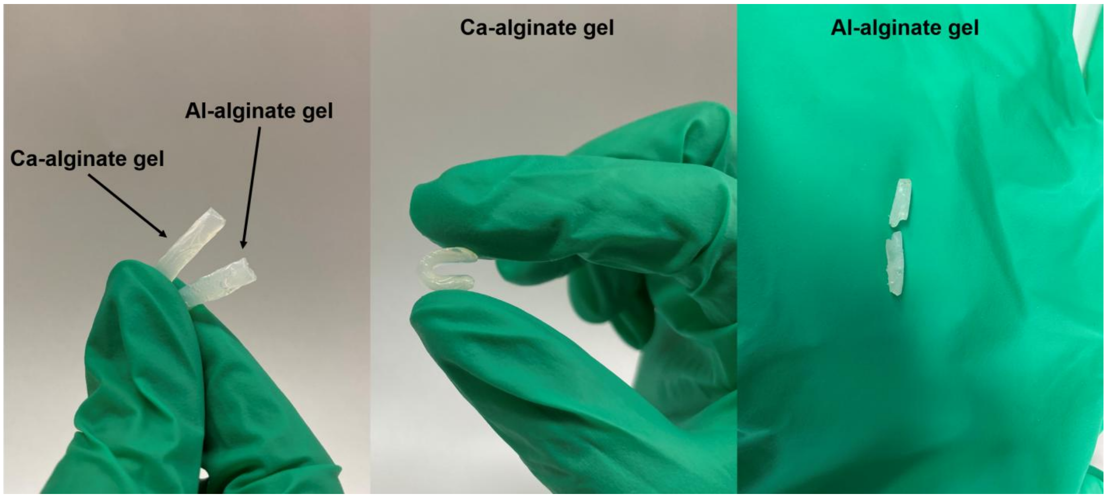

Supplementary Figure 9. Comparison of the mechanical properties of different alginate hydrogels. Hydrogels were prepared by casting the alginate sodium aqueous solution (5 wt%) onto the glass plate and coagulating in the $CaCl_2$ and $AlCl_3$ solution (5 g/L).

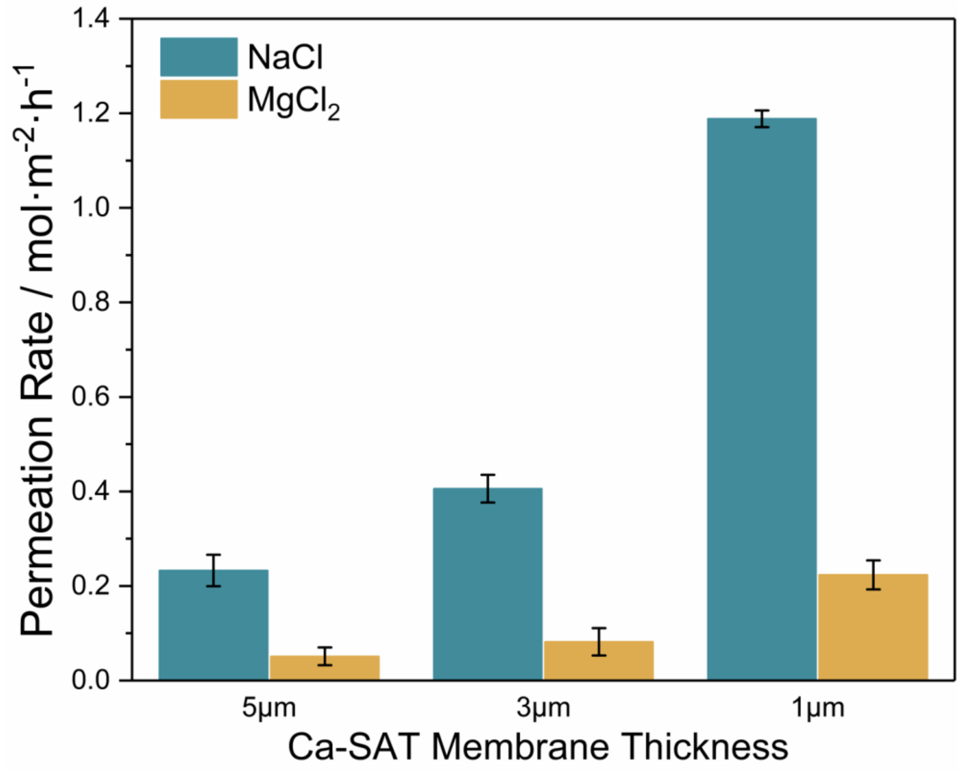

Supplementary Figure 10. Effect of membrane thickness (1, 3 and 5 μm) on ion permeation behavior through the Ca-SAT membranes. Membranes with various thicknesses were synthesized by changing the volume of filtrated nanosheet solutions. The error bars represent the standard deviations.

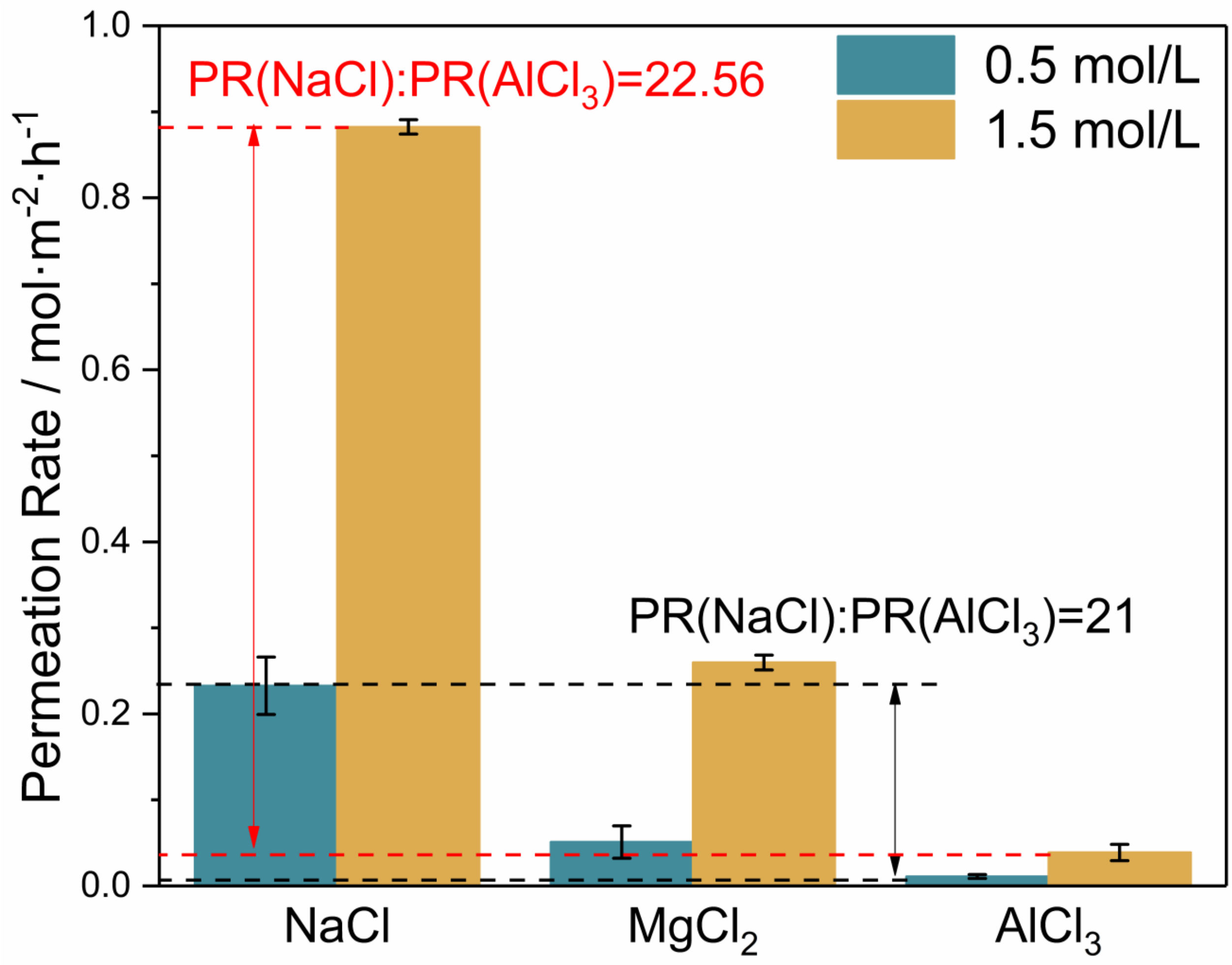

Supplementary Figure 11. Permeation rates of NaCl, $MgCl_2$ and $AlCl_3$ through the original and Ca-SAT membranes with different concentrations of 0.5 and 1.5 M. The error bars represent the standard deviations.

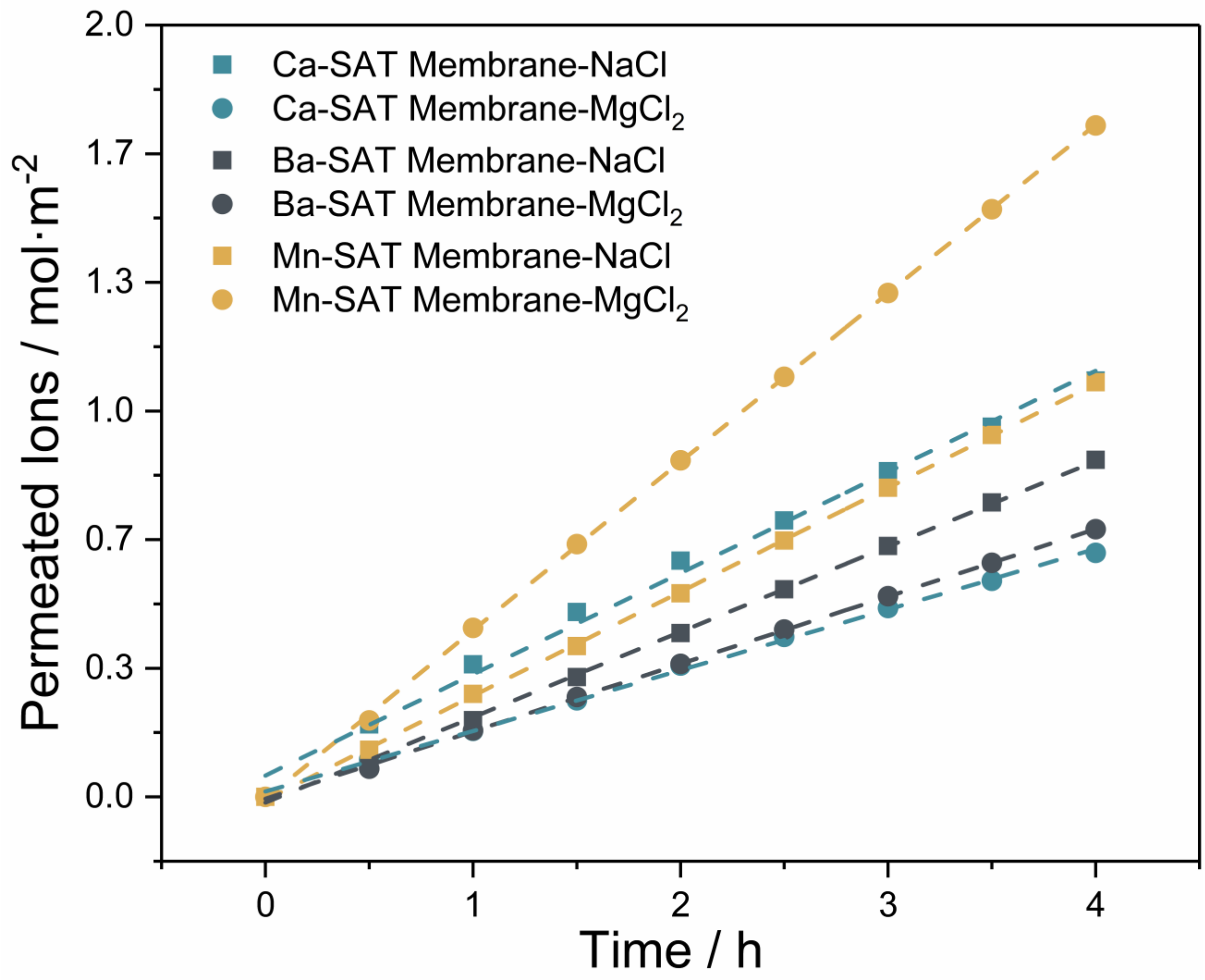

Supplementary Figure 12. Permeability over time of the SAT membrane crosslinked by Ca^{2+}, Ba^{2+} and Mn^{2+} for 0.5 M NaCl and $MgCl_2$.

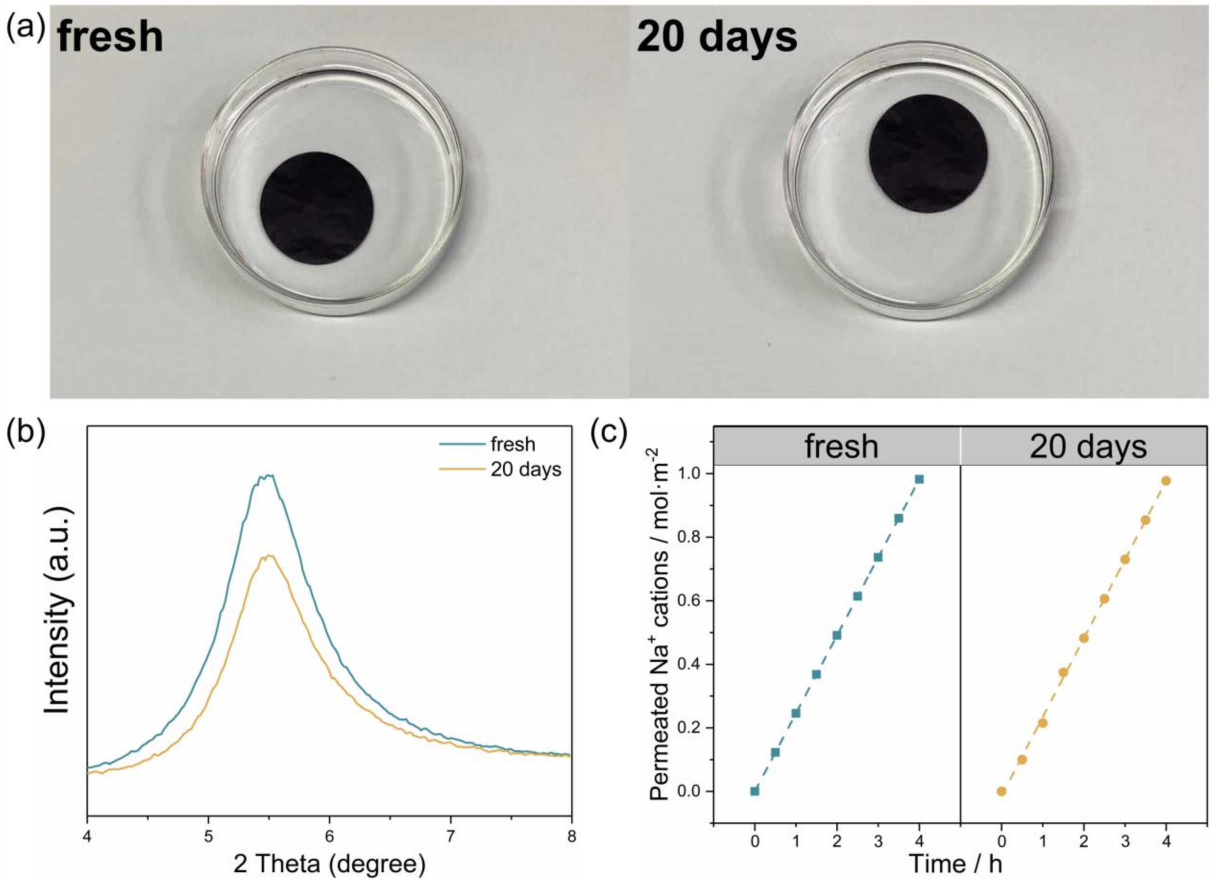

Supplementary Figure 13. Long-term immersion treatment of the Ca-SAT membrane in DI water for 20 days. (a) Photos of the Ca-SAT membrane before and after being soaked in water. (b) XRD measurement of the membrane before and after the immersion treatment. (c) 0.5 M NaCl permeation through the Ca-SAT membrane before and after the immersion treatment.

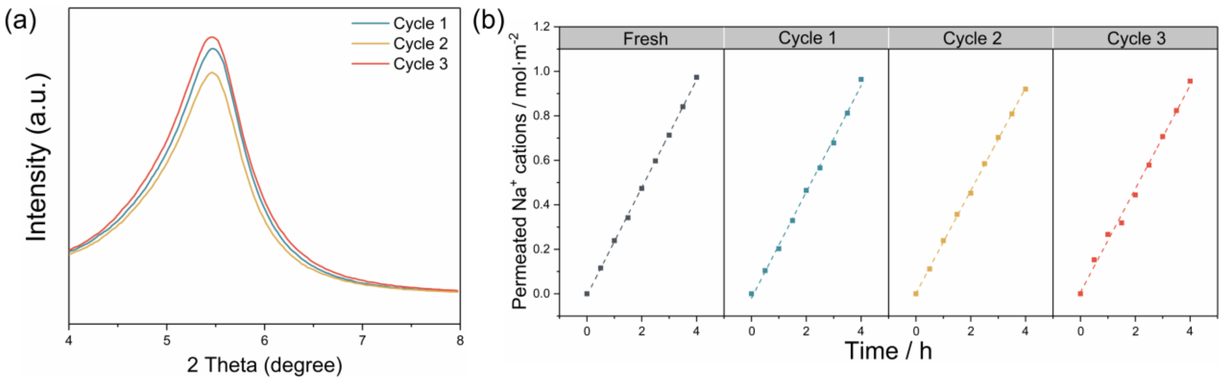

Supplementary Figure 14. Cyclic permeation/drying evaluation of the Ca-SAT membrane. (a) XRD results of the Ca-SAT membrane after the permeation tests. (b) Changes in the 0.5 M NaCl permeation rate of each cycle. After each permeation measurement, the membrane was rinsed thoroughly with DI water until the conductivity of the rinse liquid was below 0.3 μS/cm and then dried at RT for 24 h.

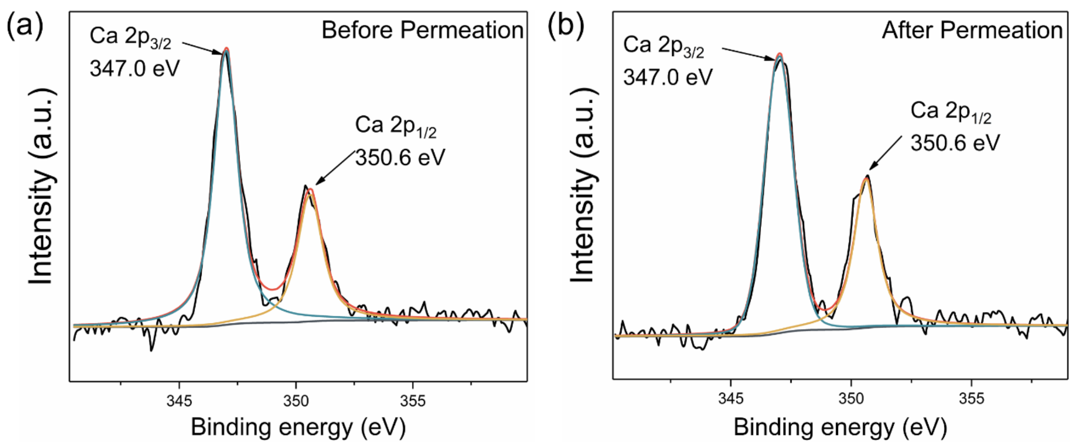

Supplementary Figure 15. XPS analysis for Ca in the Ca-SAT membrane before (a) and after (b) the 0.5 M NaCl permeation test. Before being subjected to XPS measurements, the membrane after the permeation test was rinsed thoroughly with DI water until the conductivity of the rinse liquid was below 0.3 μS/cm to eliminate the disturbance from impurities.

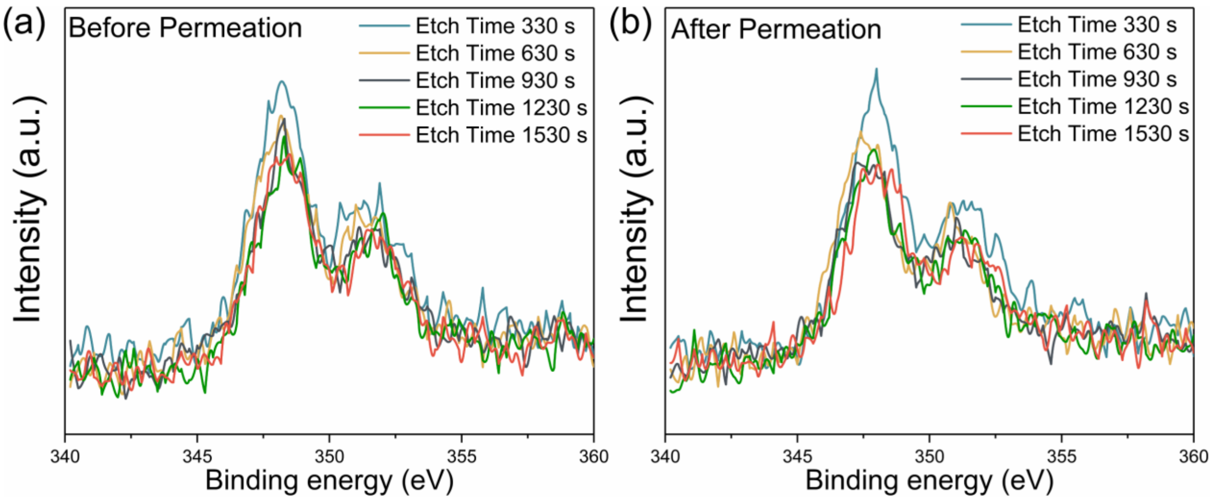

Supplementary Figure 16. XPS spectra of Ca 2p with the etching depth along the membrane thickness direction before (a) and after (b) the permeation test was performed. The etching process was conducted perpendicular to the membrane from the surface with different etching times.

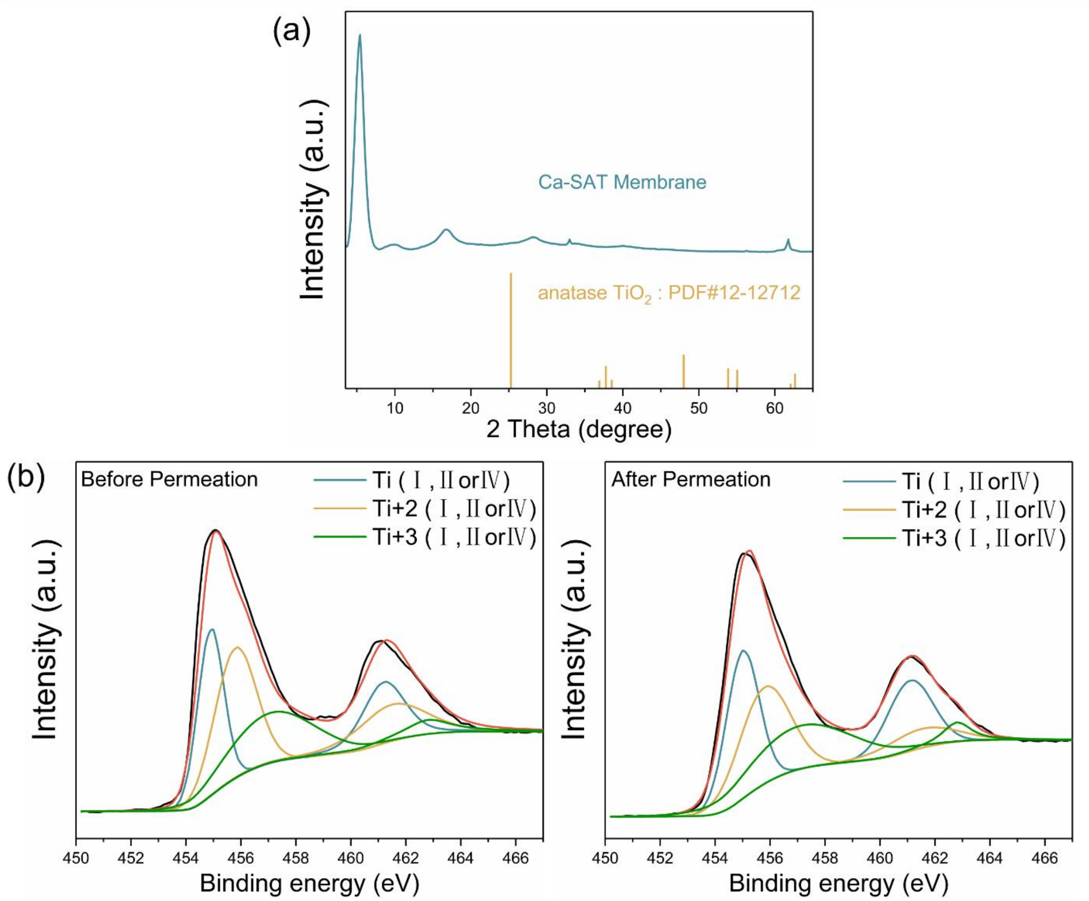

Supplementary Figure 17. Full XRD patterns (a) and high-resolution Ti 2p XPS spectra (b) of the Ca-SAT membrane before and after the permeation test.

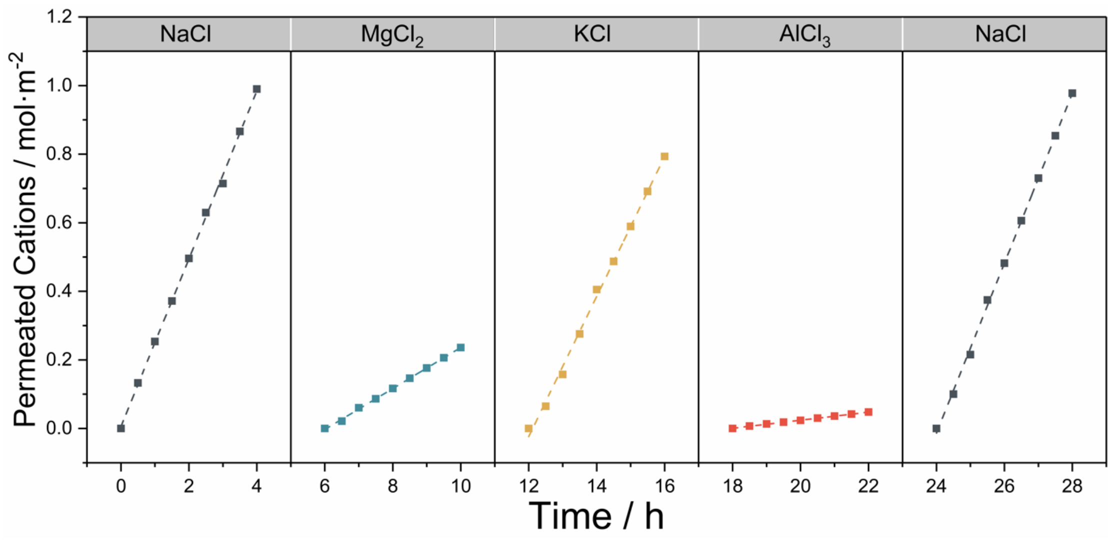

Supplementary Figure 18. Regeneration ability of the Ca-SAT membrane. After each ion permeation test, the membrane was rinsed thoroughly with DI water until the conductivity of the rinse liquid was below 0.3 μS/cm. All the concentrations of the feed solutions were 0.5 M.

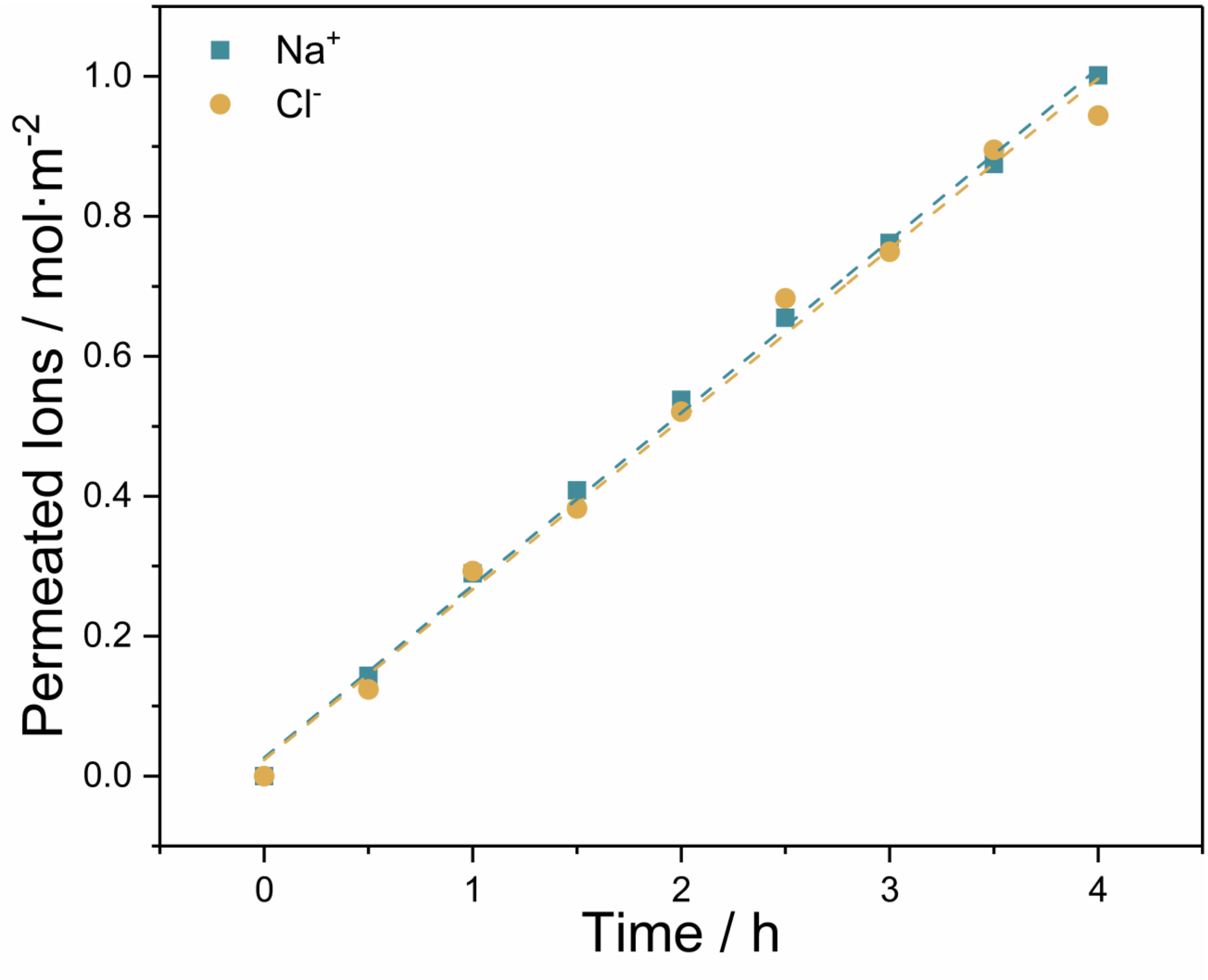

Supplementary Figure 19. Concentrations of Na+ and Cl- in the permeate compartment through the Ca-SAT membrane with 0.5 M NaCl feed solution. Cations and anions transport through the membrane in a stoichiometric manner to maintain the charge neutrality on each side, consistent with previous studies[3-4].

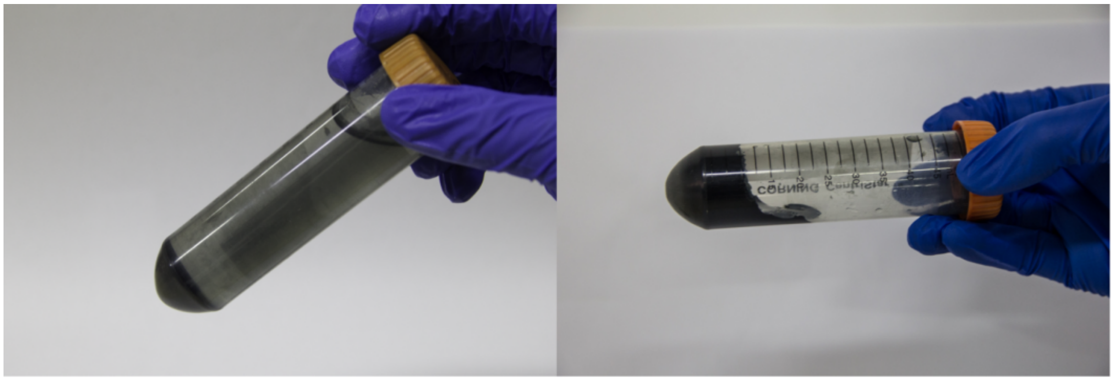

Supplementary Figure 20. Repeated centrifugation washing cycles of MILD- $Ti_3C_2T_x$. The same amount of MILD-$Ti_3C_2T_x$ powder after the first cycle (left) and seven centrifuge cycles (right) during washing showed that the sediment swelled after the seventh wash in a 50-mL centrifuge tube.

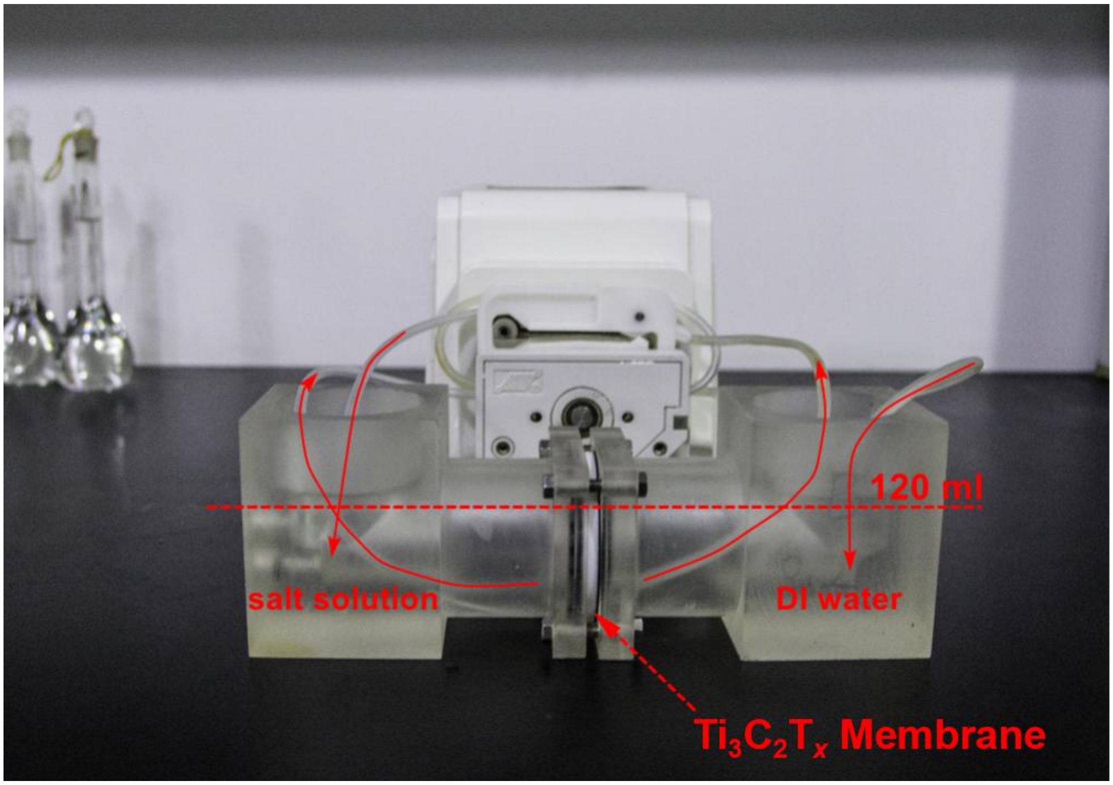

Supplementary Figure 21. Homemade U-shaped filtration device. Considering that the magnetic stirrer that was usually chosen in previous studies could not effectively avoid the concentration polarization effect[5-6], a peristaltic pump was selected to exchange the solution around the membrane surface with the bulk solution. The flow rate of the pump was set to 24 mL/min, which allowed circulation of the entire solution in each compartment in 5 min.

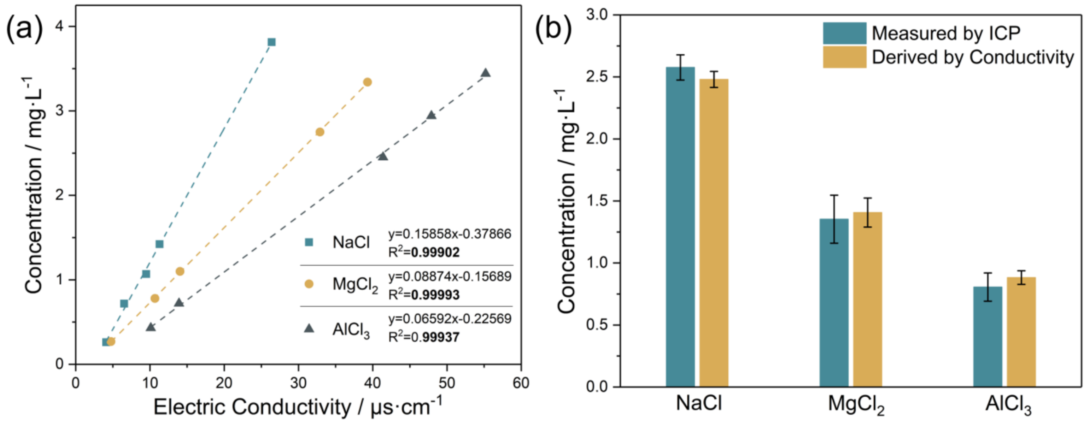

Supplementary Figure 22. Comparison of the ion concentration determined by inductively coupled plasma (ICP) mass spectrometry (MS) and solution conductivity. (a) Linear relationship between the concentration and the corresponding conductivity. (b) Ion concentration in the permeate compartment determined by ICP-MS and solution conductivity. The error bars represent the standard deviations.

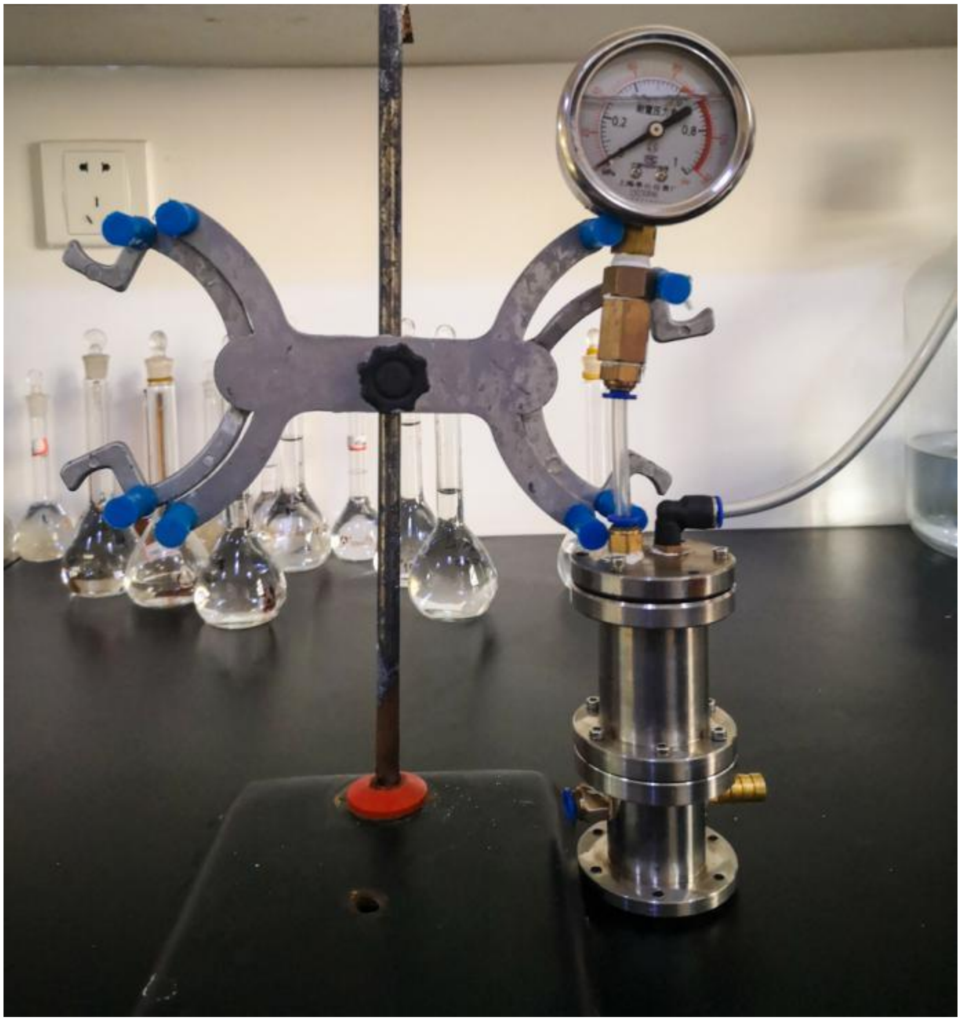

Supplementary Figure 23. Homemade dead-end filtration device.

Supplementary Table 1. Experimental ions radius and hydration free energy of different ions[7-8].

Hydrated mental ions	Radius (Å)	Hydrated energy (KJ/mol)
Na^{+}	3.58	-409
K^{+}	3.31	-322
Mg^{2+}	4.28	-1921
Fe^{2+}	4.57	-4430
Al^{3+}	4.80	-4665

Supplementary Table 2. A contrastive study between our membranes (Ca-SAT membrane) and some reported polymer-based ion-exchange membrane.

Membrane	U_{H+}(10^{-3}m h^{-1})	$S_{(H+/Fe2+)}$	Ref.
**PI membranes**	6<br>8.7<br>11	16<br>16.3<br>17	9
**Mixed charge PPO AEMs**	5	12	10
**Quaternized PPO based hybrid membranes**	5	17	11
**PVA and glycidyltrimethyl ammonium chloride (EPTAC) blending membranes**	11<br>12.3<br>13	21.5<br>20<br>17.8	12
**PVA and multi-alkoxy silicon copolymer blending membranes**	10	24	13
**DF-120 commercial membrane**	8.5	18.5	12
**QPAE-Br membranes**	10	3.5	14
**PVA treated with alkoxysilanes membranes**	8<br>10	15.9<br>21	15
**QPAES-DBs membranes**	5.06	3.89	16
**Ca-SAT membrane (5μm, this work)**	7.22	44.57	\

Supplementary Table 3. Na_2SO_4 rejection comparisons of Mn-SAT membranes in this work with other membranes in literature.

Membrane	Thickness	Zeta potential (mV)	Feed concentration	Pressure (bar)	Permeance	Na_2SO_4 Rejection	Ref.
**TMC cross-linked GO/PSF**	14	-41	10 mM	3.4	8	46	17
**Based-refluxing reduced GO/PVDF**	53	\	20 mM	1	3.26	60	18
**G-CNTm/PVDF**	40	-103	10 mM	5	11.33	81	19
**GO&EDA_HPEI 60K/PDA-PC**	69.4	-52	1000 ppm	1	5	38	20
**GO/PSf**	150	\	2000 ppm	15	11	65	21
**GO/Cellulose**	200	\	10 mM	\	8	67	22
**GO/PVDF**	50	\	3 mM	4	2.4	79.5	23
**GO@PAN**	128	\	\	1	1.8	56.7	24
**TMPyP/GO/PC**	\	\	2000	8	11.6	88	25

**PEI/GO/h-PAN**	77	-30	500 ppm	4	16.4	86.76	24
**GO/PAH/h-PAN**	37	\	6.7 mM	6.9	2	68	27
**Silica/polypiperazine amide/PES**	42	\	2000 ppm	6	7.8	97.3	28
**mMSN/PA**	100	\	5 mM	6	5.4	80	29
**MWCNT-OH TFN**	77	\			6.9	97.6	30
**MWCNT-COOH TFN**	84	\	2000 ppm	6	6.2	96.6	31
**MWCNT-NH TFN**	71	\			5.3	96.8	32
**Commercial DK membrane**	300	-45	50 ppm	2	7.3	93.2	31-32
**Commercial DL membrane**	700	-60	50 ppm	2	6.2	95.8	31-32
**GO embedded mixed matrix membrane**	\	\	1000	10	5	72	33
**GO embedded PA Membrane**	\	\	2000	15	1.53	96	34

**Mn-SAT membrane (50 nm, this work)**	50	-3.7	50 ppm	2	16.5	84	\
**Mn-SAT membrane (80 nm, this work)**	70	-3.7	50 ppm	2	12.7	100	\

Supplementary References

[1] Zhang, C. et al. Oxidation stability of colloidal two-dimensional titanium carbides (MXenes). *Chem. Mater.* **29,** 4848-4856, (2017).

[2] Alhabeb, M. et al. Guidelines for synthesis and processing of two-dimensional titanium carbide ($Ti_3C_2T_x$ MXene). *Chem. Mater.* **29,** 7633−7644, (2017**).**

[3] Ding, L. et al. Effective ion sieving with $Ti_3C_2T_x$ MXene membranes for production of drinking water from seawater. *Nat. Sustain.* **3,** 296-302, (2020)

[4] Joshi, R. et al. Precise and ultrafast molecular sieving through graphene oxide membranes. *Science* **343,** 752-754, (2014).

[5] Ren, C., Hatzell, K., Alhabeb, M., Zhang, L., Mahmoud, K. A., Gogotsi Y. Charge- and size-selective ion sieving through $Ti_3C_2T_x$ MXene membranes. *Phys. Chem. Lett.* **6,** 4026-4031, (2015).

[6] Lu, S., Wei, Y., Deng, J., Ding, L., Li, Z., Wang, H. Self-crosslinked MXene ($Ti_3C_2T_x$) membranes with good antiswelling property for monovalent metal ion exclusion. *ACS Nano* **13,** 10535-10544, (2019).

[7] Marcus, Y. Thermodynamics of solvation of ions part 5-gibbs free energy of hydration at 298.15 K. *J. Chem. Soc. Faraday trans.* **87,** 2995-2999, (1991).

[8] Michael, D. T. et al. The proton's absolute aqueous enthalpy and gibbs free energy of solvation from cluster-ion solvation data. *J. Phys. Chem. A* **102,** 7787-7794, (1998).

[9] Khan, M. I. et al. Mustaqeem M BPPO-Based anion exchange membranes for acid recovery via diffusion dialysis. *Materials.* **10,** 266, (2017).

[10] Wang, L. et al. Mixed-charge poly(2,6-dimethyl-phenylene oxide) anion exchange membrane for diffusion dialysis in acid recovery. *J. Membr. Sci.* **549,** 543-549, (2017).

[11] Luo, J., Wu, C., Wu, Y., Xu, T. Diffusion dialysis of hydrochloride acid at different temperatures using PPO–SiO2 hybrid anion exchange membranes. *J. Membr. Sci.* **247,** 240-249, (2010).

[12] Cheng, C., Yang, Z., Pan, J., Tong, B., Xu, T. Facile and cost effective PVA based hybrid membrane fabrication for acid recovery. *J. Sep. Pur.* **136,** 250-257, (2014).

[13] Wu, C., Wu, Y., Luo, J., Xu, T., Fu, Y. Anion-exchange hybrid membranes from PVA and multi-alkoxy silicon copolymer tailored for diffusion dialysis process. *J. Membr. Sci.* **356,** 96-104, (2010).

[14] Yue, X., Wu, W., Chen, G.,Yang, C., Liao, S., Li, X. Influence of 2, 2′, 6, 6′-tetramethyl biphenol-based anion-exchange membranes on the diffusion dialysis of hydrochloride acid. *J. Appl. Polym. Sci.* **134,** 45333, (2017).

[15] Wu, Y., Wu, C., Li, Y., Xu, T., Fu, Y. PVA-Silica anion-exchange hybrid membranes prepared through a aopolymer crosslinking agent. *J. Membr. Sci.* **350,** 322-332, (2010).

[16] Feng, J., Chen, J., Wei, B., Liao, S., Yu, Y., Li, X. Series-connected hexacations crosslinked anion exchange membranes for diffusion dialysis in acid recovery. *J. Membr. Sci.* **570,** 120-129, (2019).

[17] Hu, M, Mi, B. Enabling graphene oxide nanosheets as water separation membranes. *Environ. Sci. Technol.* **47,** 3715-3723, (2013).

[18] Han, Y., Xu, Z., Gao, C. Ultrathin graphene nanofiltration membrane for water purification. *Adv. Funct. Mater.* **23,** 3693-3700, (2013).

[19] Han, Y., Jiang, Y., Gao, C. High-flux graphene oxide nanofiltration membrane intercalated by carbon nanotubes. *ACS Appl. Mater. Inter.* **7,** 8147-8155, (2015).

[20] Zhang, Y., Zhang, S., Chung, T. Nanometric graphene oxide framework membranes with enhanced heavy metal removal via nanofiltration. *Environ. Sci. Technol.* **49,** 10235-10242, (2015).

[21] Wei, Y., Zhang, Y., Gao, X., Yuan, Y., Su, B., Gao, C. Declining flux and narrowing nanochannels under wrinkles of compacted graphene oxide nanofiltration membranes. *Carbon* **108,** 568-575, (2016).

[22] Liu, G., Ye, H., Li, A. Graphene oxide for high-efficiency separation membranes: Role of electrostatic interactions. *Carbon* **110,** 56-61, (2016).

[23] Song, Y., Li, T., Zhou, J., Li, Z., Gao, C. Analysis of nanofiltration membrane performance during softening process of simulated brackish groundwater. *Desalination* **399,** 40-46, (2016).

[24] Wang, J. et al. Graphene oxide as an effective barrier on a porous nanofibrous membrane for water treatment. *ACS Appl. Mater. Inter.* **8,** 6211-6218, (2016).

[25] Xu, X., Lin, L., Du, D., Zhang, X., Wu, J., Xu, Z. Graphene oxide nanofiltration membranes stabilized by cationic porphyrin for high salt rejection. *ACS Appl. Mater. Inter.* **8,** 12588-12593, (2016).

[26] Wang, T., Lu, J., Mao, L., Wang, Z. Electric field assisted layer-by-layer assembly of graphene oxide containing nanofiltration membrane. *J. Membr. Sci.* **515,** 125-133, (2016).

[27] Oh, Y. et al. Understanding the pH-responsive behavior of graphene oxide membrane in removing ions and organic micropollutants. *J. Membr. Sci.* **541,** 235-243, (2017).

[28] Hu, D., Xu, Z., Chen, C. Polypiperazine-amide nanofiltration membrane containing silica nanoparticles prepared by interfacial polymerization. *Desalination* **301,** 75-81, (2012).

[29] Wu, H., Tang, B., Wu, P. Optimizing polyamide thin film composite membrane covalently bonded with modified mesoporous silica nanoparticles. *J. Membr. Sci.* **428,** 341-348, (2013).

[30] Xue, S., Xu, Z., Tang, Y., Ji, C. Polypiperazine-amide nanofiltration membrane modified by different functionalized multiwalled carbon nanotubes (MWCNTs). *ACS Appl. Mater. Inter.* **8,** 19135-19144, (2016).

[31] Boya, X. The development of carboxylic acid separation by nanofiltration membrane for carboxylate platform using lingnocellulosic biomass. *The Pennsylvania State University* 53-55, (2014).

[32] Hafedh, S., Nihel, B. A., John, P., André, D. Interplay between the transport of solutes across nanofiltration membranes and the thermal properties of the thin active layer. *Langmuir* **26,** 2574-2583, (2010).

[33] Bano, S., Mahmood, A., Kim, S. J., Lee, K. H. Graphene oxide modified polyamide nanofiltration membrane with improved flux and antifouling properties. *J. Mater. Chem. A.* **3,** 2065-2071, (2015).

[34] Ganesh, B. M., Isloor, A. M., Ismail, A. F. enhanced hydrophilicity and salt rejection study of graphene oxide-polysulfone mixed matrix membrane. *Desalination* **313,** 199-207, (2013).
