# Supplementary material for: Ion sieving by a two-dimensional Ti3C2Tx alginate lamellar membrane with stable interlayer spacing
Source: Nat Commun. 2020 Jul 15;11:3540. doi: 10.1038/s41467-020-17373-4 (PMC7363915; doi:10.1038/s41467-020-17373-4)
Supplement: Supplementary file 2 — Peer Review File [file 41467_2020_17373_MOESM2_ESM.docx]

Peer Review Information

**Journal:** Nature Communications

**Manuscript title:** Ion Sieving by the 2D Ti_3_C_2_T*_x_*-Alginate Lamellar Membrane with Stable Interlayer Spacing

**Corresponding author name(s):** Prof Lei Wang

**Editorial notes:**

**none**

**Reviewer comments & decisions:**

**Reviewer comments, first version:**

Reviewer #1:
Remarks to the Author:
The authors have properly addressed all the issues which were araised by reviewers, therefore it can be recommend for publication in the present form.


Reviewer #2:
Remarks to the Author:
In this work, Wang et al. designed Ca-alginate pillared Ti3C2Tx with stable laminar architecture. After pillaring, the Ti3C2Tx membrane presents outstanding sieving property towards cations. However, more additional evidences should be provided to strongly support the author's claim. Therefore, I cannot recommend this manuscript to be accepted at the current stage. It can be resubmitted if the following data could be provided.
The comments are as follows:
1. Long-term stability of the Ca-alginate pillared Ti3C2Tx for cations permeation is one of the important parameters for membrane performance.
2. To further confirm the stability of Ca-alginate pillared Ti3C2Tx membrane, the cycles of desalination/drying performance is essential, including the XRD results.
3. It would be better for the authors to show the results of thickness dependent permeation rate of different cations through Ca-alginate pillared Ti3C2Tx membrane.
4. The existence of Ca2+ and its current form in the Ca-alginate pillared Ti3C2Tx membrane after ion permeation needs to be confirmed.
5. XPS spectra with the corresponding depth profiles would be a good tool to confirm the concentration of Ca2+ between Ti3C2Tx layers before and after ion permeation.
6. It is well known that MXene is easy to be oxidized in the aqueous solution. Therefore, as a membrane used in aqueous solution, it is concerning that whether MXene is stable during the operation.
7. The regeneration ability of the Ca-alginate pillared Ti3C2Tx membrane also needs to be investigated.


Reviewer #3:
Remarks to the Author:
The authors have reported a lamellar membrane based on 2D Ti3C2Tx MXene that shows promising ion-sieving properties. There is a growing interest in the study of 2D MXenes as high performance membranes for ion-sieving and water desalination. The authors have presented reasonable data. The overall concept, however, of hybridization of MXenes with alginates and creating a lamellar out of them with varying ions is not novel and despite promising performances for the specific application of ion-sieving, do not show a step forward in the field. Nevertheless, the paper might be of interest to readers of Nature communications if the paper is significantly improved in a major revision, more experiments are performed My comments and questions to the authors are as follows:
- The language needs to be corrected by a native English speaking person, as it contains just too many grammatical errors (e.g. ” and after assembled by van der Waals force”, or ” Despite several approaches have been proposed to restrict ” is incorrect English). In other case it cannot be accepted.
- The “n” in MXene formula (line 59, page 3) can also be 4, I suggest that authors update and correct this in their manuscript.
- After modification of MXene surfaces with sodium alginate, and incorporation of cation-pillars, the d-spacing shows smaller changes when soaked in water and various salt solutions, however, it still varies around ~0.1 nm. Could the authors explain why?
- Why does the membrane structure become brittle after pillaring with Al3+, the authors need to explain this.
- In figure 4e, why does the permeation rate for same Na cations with different anions differ that much? What is the role of anions in salt solutions in ion-sieving behavior of MXene membranes? It is also known that in aqueous solutions, some anions can actually (electro)chemically intercalate between MXene layers. Have authors considered this?
- Why is the intercalation/diffusion speed of Al3+ is that slow compared to all other ions despite the large interlayer spacing for the alginate-pillared membranes? The large interlayer spacing should significantly reduce the interaction and diffusion barrier for intercalation of high charge density Al3+ cations in the MXene membranes. This point needs a clear explanation, ideally supported by some experiments.
- The authors do not discuss the effect of membrane thickness on the performance at all. That is an important parameter in the ion movement and interactions for MXene membranes. The authors need to show at least some experiment about this relation.
- The authors indicate that they dry their membrane at RT. However, it is known that MXene membranes do not completely dry at RT even under vacuum. Some of the interlayer water even stay in MXene films after drying at temperatures higher than 500 C. Considering this, how can they claim that different cation-pillars in their alginate hybrid membranes are the only factor affecting the d-spacing in their films? Each of those cations can have a different hydration shell. Also, the amount of the absorbed water (interlayer water) is known to be higher for multivalent cations such as Mg2+ and Al3+.
- A concerning point to me is about the MXene solutions the authors have made. In Figure SI 1, The UV-Vis spectra of the as prepared MXene solution is shown, which clearly shows that the Ti3C2Tx solution is highly oxidized. The broad bump at around 300 nm range are for TiO2. And from the intensity of the TiO2 peak in the overall MXene solution spectra I believe the solution authors have prepared is highly oxidized. That can have direct affect in hybridization process with alginate, as well as the performance of the membranes. I recommend either authors explain the reason for this severe oxidation in their solutions, its affect on the membranes and their processing, or try to process a new solution and suppress the oxidation of their MXenes. A normal Ti3C2Tx solution generally does not show the TiO2 bump or it should be very negligible. The minimum thing the authors must do is to show data for a non-oxidized MXene, and its film to prove either that its ion-sieving properties are similar to the oxidized, or different.

**Author rebuttal, first version:**

**General Comment**
*The authors have properly addressed all the issues which were araised by reviewers, therefore it can be recommend for publication in the present form.*

**Response to the General Comment**

We thank the reviewer very much for the positive evaluation of our work.

**Reviewer #2**

**General Comment**

*In this work, Wang et al. designed Ca-alginate pillared Ti3C2Tx with stable laminar architecture. After pillaring, the Ti3C2Tx membrane presents outstanding sieving property towards cations. However, more additional evidences should be provided to strongly support the author's claim. Therefore, I cannot recommend this manuscript to be accepted at the current stage. It can be resubmitted if the following data could be provided.*

**Response to the General Comment**

We are grateful for the positive evaluation of our work. All the revised parts can be found in the revised manuscript with changes marked.

**Comment 1**

*Long-term stability of the Ca-alginate pillared Ti3C2Tx for cations permeation is one of the important parameters for membrane performance.*

**Response to Comment 1**

We thank the reviewer for the kind suggestion. We agree that the long-term stability is indeed a critical issue for developing a high-performance membrane, and several additional experiments were supplemented to confirm the structure and performance stability. The Ca-alginate pillared Ti_3_C_2_T*_x_* membrane was resubjected for long-term immersion in DI water, after which the dissolution of the membrane and the changes in the fixed d-spacing were tested. Moreover, ion permeation in the immersion-treated Ca-SAT membrane was also investigated. The experimental results confirm the excellent structural stability and durability of the Ca-SAT membrane in aqueous environments. A detailed description of the stability experiments (Line 267, Page 13) has been added to the revised manuscript and the Supplementary Materials as follows:

“Structural stability is a critical parameter in practical ion-sieving applications; in particular, the laminate structure of the membrane was adjusted with angstrom-scale precision in this study. To provide a comprehensive evaluation of the stability of pillared Ti_3_C_2_T*_x_* membranes, the as-prepared Ca-SAT membrane was subjected to a long-term immersion treatment. The membrane exhibited no apparent sign of disintegration or dissolution in DI water even after 20 days (Supplementary Fig. 13 (a)). The corresponding XRD results confirmed the consistent interlayer spacing and suggested that the stabilized structure was not affected by long-term immersion (Supplementary Fig. 13 (b)). It is also clear that the Na^+^ permeation rate through the Ca-SAT membrane was constant before and after the immersion treatment (Supplementary Fig. 13 (c)), implying that the ion-sieving performance would not deteriorate by long-term operation in an aqueous environment.

**Supplementary Figure 13. Long-term immersion of the Ca-SAT membrane in DI water for 20 days.** (a) Photos of the Ca-SAT membrane before and after being soaked in water. (b) XRD measurement of the membrane before and after the immersion treatment. (c) 0.5 M NaCl permeation through the Ca-SAT membrane before and after the immersion treatment.”

**Comment 2**

*To further confirm the stability of Ca-alginate pillared Ti3C2Tx membrane, the cycles of desalination/drying performance is essential, including the XRD results.*

**Response to Comment 2**

We thank the reviewer for the kind reminder. We have employed experiments to confirm the changes in both lamellar structure and permeation performance during the diffusion/drying cycles. The measurement results indicated the excellent durability of our Ca-SAT membrane. Detailed conditions and results have been added to the revised manuscript (Line 276, Page 13) and the Supplementary Materials as follows:

“Furthermore, during 3 permeation/drying cycles, ion permeation through the Ca-SAT membrane remained at a substantially constant rate (Supplementary Fig. 14). The XRD measurement results also certified that the stable lamellar structure could withstand the cyclic permeation/drying test and that the pillared Ti_3_C_2_T*_x_* membrane demonstrated excellent structural durability.

**Supplementary Figure 14. Cyclic permeation/drying evaluation of the Ca-SAT membrane.** (a) XRD results of the Ca-SAT membrane after the permeation tests. (b) Changes in the 0.5 M NaCl permeation rate for each cycle. After each permeation measurement, the membrane was rinsed thoroughly with DI water until the conductivity of the rinse liquid was below 0.3 μS/cm and then dried at RT for 24 h.”

**Comment 3**

*It would be better for the authors to show the results of thickness dependent permeation rate of different cations through Ca-alginate pillared Ti3C2Tx membrane.*

**Response to Comment 3**

We thank the reviewer for the kind suggestion. A study on the dependence of the cation permeation rate on Ca-SAT membrane thickness (1 μm, 3 μm, 5 μm) was conducted. The permeation rate exhibited an apparent increase with decreasing thickness, while the selectivity dependent on the valence of cations almost did not change. The experimental data and discussion have been added in the revised manuscript (Line 233, Page 11) and the Supplementary Materials as below:

“We further studied the dependence of the cation PRs on the thickness of the Ca-SAT membrane (Supplementary Fig. 10). The ion PRs exhibited a distinct decrease with increasing thickness, which could be attributed to the prolonged permeation length. Nevertheless, we also observed that the ion-sieving property of the Ca-SAT membrane was dependent on the cation valence and was almost unaffected by the thickness variation.

**Supplementary Figure 10. Effect of membrane thickness (1, 3 and 5 μm) on ion permeation behavior through the Ca-SAT membranes.** Membranes with various thicknesses were synthesized by changing the volume of filtrated nanosheet solutions.”

**Comment 4**

*The existence of Ca2+ and its current form in the Ca-alginate pillared Ti3C2Tx membrane after ion permeation needs to be confirmed.*

**Response to Comment 4**

We thank the reviewer for the kind comment. As suggested, XPS measurements of the Ca-SAT membrane before and after the ion permeation test were conducted. We found that the Ca 2p spectrum of the membrane was consistent with the typical Ca 2p spectrum of Ca-alginate gel (*Zhang M. J. et al., Water Research, 2018, 129, 337-346;* and *Guo Z. W. et al., Journal of Colloid and Interface Science, 2020, 562, 224-234.*), indicating the crosslinking reactions between Ca^2+^ and alginate molecules. The XPS results also indicated that the form of Ca remained the same and that the Ca-alginate pillars were rigidly fixed in the Ti_3_C_2_T*_x_* membrane during the test process in the aqueous environment. Detailed results and explanations have been added to the revised manuscript (Line 280, Page 13) and the Supplementary Materials as follows:

“To further confirm the variation in the forms of Ca that existed and the concentration in the Ca-SAT membrane after ion permeation, XPS measurements were conducted. Consistent with the typical XPS spectrum of Ca-alginate gel in previous reports,^48-49^ the Ca 2p region could be fitted by two peaks at binding energies of 350.6 and 347.0 eV, which suggested the crosslinking interaction between Ca^2+^ and alginate molecules (Supplementary Fig. 15). Moreover, the unchanged binding energies of Ca 2p peaks after ion permeation illustrated that no changes in Ca occurred, and the Ca-alginate pillars were firmly anchored in the interlayer spacing.

**Ref. 48:** Zhang, M. J.; Hong, H. C.; Lin, H. J.; Shen, L. G.; Yu, H. Y.; Ma, G. C.; Chen, J. R.; Liao, B. Q. Mechanistic insights into alginate fouling caused by calcium ions based on terahertz time-domain spectra analyses and DFT calculations. Water Res. 2018, 129, 337-346.

**Ref. 49:** Guo, Z. W.; Li, Q.; Li, Z. Y.; Liu, C.; Liu, X. R.; Liu, Y. Y.; Dong, G. L.; Lan, T.; Wei, Y. Fabrication of efficient alginate composite beads embedded with N-doped carbon dots and their application for enhanced rare earth elements adsorption from aqueous solutions. J. Colloid Interface Sci. 2020, 562, 224–234.

**Supplementary Figure 15. XPS analysis of Ca in the Ca-SAT membrane before (a) and after (b) the 0.5 M NaCl permeation test.** After the permeation test, the membrane was rinsed thoroughly with DI water until the conductivity of the rinse liquid was below 0.3 μS/cm to eliminate the disturbance from impurities before being subjected to XPS measurements.”

**Comment 5**

*XPS spectra with the corresponding depth profiles would be a good tool to confirm the concentration of Ca2+ between Ti3C2Tx layers before and after ion permeation.*

**Response to Comment 5**

Thank you for your kind reminder. To test the Ca concentration changes in the interlayer before and after the permeation test, XPS measurements with different etching depths along the membrane thickness direction were conducted. We confirmed that the Ca concentration was consistent during the permeation test, which suggested that the Ca-alginate pillar was not impaired during the ion permeation process. The detailed data were added to the revised manuscript (Line 287, Page 14) and the Supplementary Materials as follows:

“In addition, according to the Ca depth profiles along the membrane thickness direction, the concentration of Ca contained in the membrane was basically unchanged before and after ion permeation, suggesting that the Ca-alginate pillar was not affected during the ion-sieving process (Supplementary Fig. 16). The constant Ca concentration with different depths also confirmed that the hydrogel pillars were homogeneously distributed in the membrane, consistent with the SEM result (Fig. 2 (f)).

**Supplementary Figure 16. XPS spectra of Ca 2p with the etching depth along the membrane thickness direction before (a) and after (b) the permeation test was performed.** The etching process was conducted perpendicular to the membrane from the surface with different etching times.”

**Comment 6**

*It is well known that MXene is easy to be oxidized in the aqueous solution. Therefore, as a membrane used in aqueous solution, it is concerning that whether MXene is stable during the operation.*

**Response to Comment 6**

We thank the reviewer for the kind comment. The full XRD pattern of the Ca-SAT membrane after the ion permeation experiments was measured, and we confirmed that TiO_2_ anatase, which is the main oxidation product, was not formed during the experiments (*Li Z. et al., Materials Science and Engineering B, 2015, 191, 33-40;* and *Haihui W. et al., ACS Nano, 2019, 13, 10535-10544.*). Furthermore, the variation in Ti before and after ion permeation was also confirmed by XPS. To maintain measurement consistency, the membrane before the ion permeation test was resubjected to XPS characterization (Supplementary Fig. 6). From the high-resolution XPS spectra in the Ti 2p region, we observed that the proportion of TiO_2_ in the as-prepared Ca-SAT membrane was negligible, and the Ti existence form also remained the same, indicating that the membrane oxidation was slight (*Haihui W. et al., ACS Nano, 2019, 13, 10535-10544.*). Detailed data and analysis have been added to the revised manuscript (Line 292, Page 14) and the Supplementary Materials as follows:

“The full XRD patterns of the Ca-SAT membrane after the permeation test confirmed that TiO_2_ anatase, which is the main oxidation product, was not formed during operation of the membrane (Supplementary Fig. 17).^50^ The high-resolution XPS spectra of Ti 2p also demonstrate that the proportion of TiO_2_ in the as-prepared Ca-SAT membrane was negligible, and the Ti form also remained constant, indicating that the membrane oxidation was slight.^51^

**Ref. 50**: Li, Z. T.; Wang, L. B.; Sun, D. D.; Zhang, Y. D.; Liu, B. Z.; Hu, Q. K.; Zhou, A. G. Synthesis and thermal stability of two-dimensional carbide MXene Ti_3_C_2_. Mater. Sci. Eng., B 2015, 191, 33-40.

**Ref. 51**: Lu, Z.; Wei, Y. Y.; Deng, J. J.; Ding, L.; Li, Z. K.; Wang, H. H. Self-Crosslinked MXene (Ti_3_C_2_T*_x_*) Membranes with Good Antiswelling Property for Monovalent Metal Ion Exclusion. ACS Nano 2019, 13, 10535-10544.

**Supplementary Figure 17.** Full XRD patterns (a) and high-resolution Ti 2p XPS spectra (b) of the Ca-SAT membrane before and after the permeation test.”

**Comment 7**

*The regeneration ability of the Ca-alginate pillared Ti3C2Tx membrane also needs to be investigated.*

**Response to Comment 7**

We thank the reviewer for the kind suggestion. Although we have confirmed that when the two cations in the permeation tests were cycled repeatedly, the former permeation rate had little impact on the latter rate. To further assess the regeneration ability of the membrane between the two Na^+^ permeation measurements, various different cation permeations were continuously performed on the same Ca-SAT membrane. We observed that even though the membrane was subjected to a much more complete permeation process, the Na^+^ permeation rate could still be restored only by a thorough rinsing step using DI water, and the pillared membrane exhibited an excellent regeneration ability. Moreover, recent studies (*Rasool, K. et al. Scientific Reports, 2017, 7, 1598*; and *Haihui W. et al., Nature Sustainability, 2020,* 3, 296-302.) have also confirmed the outstanding antibacterial efficiency and chlorine tolerance of the Ti_3_C_2_T*_x_* membrane, which also provides evidence of the excellent regenerability of the Ti_3_C_2_T*_x_*-based membrane. The detailed data and discussion have been added in the revised manuscript (Line 296, Page 14) and the Supplementary Materials as follows:

“The regeneration ability of the Ca-SAT membrane was also evaluated, as shown in Supplementary Fig. 18. Although permeation experiments with different cations were continuously performed on the same Ca-SAT membrane after the Na^+^ permeation test was first conducted, the permeation rate of Na^+^ could be effectively restored only by thorough rinsing using DI water, indicating excellent regenerability. Moreover, recent studies have also confirmed the outstanding antibacterial efficiency and chlorine tolerance of the Ti_3_C_2_T*_x_* membrane,^52-53^ which also provides evidence of the excellent regenerability of the Ti_3_C_2_T*_x_*-based membrane.

**Ref 52**: Rasool, K.; Mahmoud, K.; Johnson, D. J.; Helal, M.; Brediyorov, G. R.; Gogotsi, Y. Efficient Antibacterial Membrane based on Two-Dimensional Ti_3_C_2_T*_x_* (MXene) Nanosheets. Sci. Rep. 2017, 7, 1598.

**Ref 53**: Ding, L.; Li, L. B.; Liu, Y. C.; Wu, Y.; Lu, Z.; Deng, J. J.; Wei, Y. Y.; Caro, J.; Wang, H. H. Effective ion sieving with Ti_3_C_2_T*_x_* MXene membranes for production of drinking water from seawater. Nat. Sustain. 2020, 3, 296-302.

**Supplementary Figure 18.** **Regeneration ability of the Ca-SAT membrane.** After each ion permeation test, the membrane was rinsed thoroughly with DI water until the conductivity of the rinse liquid was below 0.3 μS/cm. All the concentrations of the feed solutions were 0.5 M.”

**Reviewer #3**

**General Comment**

*The authors have reported a lamellar membrane based on 2D Ti3C2Tx MXene that shows promising ion-sieving properties. There is a growing interest in the study of 2D MXenes as high performance membranes for ion-sieving and water desalination. The authors have presented reasonable data. The overall concept, however, of hybridization of MXenes with alginates and creating a lamellar out of them with varying ions is not novel and despite promising performances for the specific application of ion-sieving, do not show a step forward in the field. Nevertheless, the paper might be of interest to readers of Nature communications if the paper is significantly improved in a major revision, more experiments are performed My comments and questions to the authors are as follows:*

**Response to the General Comment**

We greatly appreciate the reviewer’s positive comments. All the revised text can be found in the manuscript with changes marked.

**Comment 1**

*The language needs to be corrected by a native English speaking person, as it contains just too many grammatical errors (e.g. ”and after assembled by van der Waals force”, or ” Despite several approaches have been proposed to restrict ” is incorrect English). In other case it cannot be accepted.*

**Response to Comment 1:**

We thank the reviewer for the kind suggestion. We have improved the English of the manuscript using the language service recommended by ACS, American Journal Experts (AJE). Because these changes do not affect the content or framework of the paper, for reading convenience, we have not listed the changes in the revised paper.

**Comment 2**
*The “n” in MXene formula (line 59, page 3) can also be 4, I suggest that authors update and correct this in their manuscript.*

**Response to Comment 2:**

We thank the reviewer for the kind correction. We have corrected the mistake in the revised manuscript (Line 61, Page 3).

**Comment 3**
*After modification of MXene surfaces with sodium alginate, and incorporation of cation-pillars, the d-spacing shows smaller changes when soaked in water and various salt solutions, however, it still varies around ~0.1 nm. Could the authors explain why?*

**Response to Comment 3:**

We thank the reviewer for the kind comment. Previous reports have confirmed that the intercalation of cations into Ti_3_C_2_T*_x_* induces significant structural deformation. The study on the effect of the sodium alginate (SA)/Ti_3_C_2_T*_x_* mass ratio on the structural stability (Fig. 3(e)) indicated that only a sufficient number of alginate gel pillars in the interlayer spacing could supply sufficient force from the crosslinking interactions between the alginate molecules and crosslinking cations to overcome these structural changes. When 5 wt% SA was added in the Ti_3_C_2_T*_x_* nanosheets, the d-spacing fluctuation was distinctly suppressed from being 1.79 Å while still being restricted to approximately 0.52 Å, as indicated by the reviewer. As the SA amount further increased to 10%, the fluctuation was more efficiently restricted to 0.20 nm. Reviewing the previous literature concerning the d-spacing stabilization, when the d-spacing variation was restricted within approximately 0.50 Å in different salt solutions (*Haihui W. et al., Nature Sustainability, 2020,* *3, 296-302.* d-spacing variation: 0.57 Å; and *Chen L. et al., Nature, 2017, 550, 380-383.* d-spacing variation: 0.36 Å), the unstable lamellar structure was considered to be successfully fixed because the ion-sieving property of lamellar membranes was significantly influenced. Therefore, the ion transport behavior in the membrane incorporated with 5 wt% SA was investigated in this study, although the d-spacing was not completely stabilized. We have revised the statement to clarify it (Line 193, Page 10) in the revised manuscript as follows:

“It could be concluded that when 1 wt% sodium alginate is added to the Ti_3_C_2_T*_x_* nanoflakes, the coordination interaction between the SA molecules and crosslinking cations is unable to supply sufficient force to overcome the significant intercalation-induced structural changes. When the SA amount increased to 5%, the d-spacing fluctuation was distinctly suppressed to 0.52 Å; when the SA amount was further increased to 10%, the d-spacing remained almost constant in aqueous solution, which suggested that the membrane would have a more stable structure when more pillars were incorporated in the interlayer spacing. Considering that excess SA addition could influence the uniformity of the layer structure and obstruct the pathways, sodium alginate was set at 5-10 wt% in the nanosheet in the follow-up experiment.”

**Comment 4**

*Why does the membrane structure become brittle after pillaring with Al3+, the authors need to explain this****.***

**Response to Comment 4:**

We thank the reviewer for the kind comment. A comparison was added to demonstrate the mechanical properties of hydrogels formed after the crosslinking reactions between alginate sodium and different multivalent cations. Different from the Ca-alginate gel with excellent flexibility, the Al-alginate gel was stiff and brittle, which could be attributed to the different crosslinking mechanisms of alginate sodium with trivalent Al^3+^ (*Jiri B. et al, Biomacromolecules 2017, 18, 2478-2488.*). As a result, Al-alginate gel formed in the interlayer of Ti_3_C_2_T*_x_* laminates reduced the excellent flexibility characteristics of the membrane. We have added this explanation (Line 207, Page 10) in the revised manuscript and the Supplementary Materials as follows:

“Different from the Ca-alginate gel with excellent flexibility, the Al-alginate gel was stiff but brittle (Supplementary Fig. 9), which could be attributed to the different crosslinking mechanisms^47^. Therefore, the incorporation of Al-alginate into the interlayer reduced the flexibility of the Ti_3_C_2_T*_x_* membrane, and the Al-SAT membrane is not discussed in further detail because the brittle structural characteristics are not acceptable for industrial filtration processes.

Ref. 47: Brus, J.; Urbanova, M.; Czernek, J.; Pavelkova, M.; Kubova, K.; Vyslouzil, J.; Abbrent, S.; Konefal, R.; Horsky, J.; Vetchy, D.; Vyslouzil, J.; Kulich, P. Structure and Dynamics of Alginate Gels Cross-Linked by Polyvalent Ions Probed via Solid State NMR Spectroscopy. Biomacromolecules 2017, 18, 2478−2488

**Supplementary Figure 9. Comparison of the mechanical properties of different alginate hydrogels.** Hydrogels were prepared by casting the alginate sodium aqueous solution (5 wt%) cast onto the glass plate and coagulating in the CaCl_2_ and AlCl_3_ solution (5 g/L), respectively.”

**Comment 5**
*In figure 4e, why does the permeation rate for same Na cations with different anions differ that much? What is the role of anions in salt solutions in ion-sieving behavior of MXene membranes? It is also known that in aqueous solutions, some anions can actually (electro)chemically intercalate between MXene layers. Have authors considered this?*

**Response to Comment 5:**

We thank the reviewer for the kind comment. As indicated by the reviewer, a recent study suggested that the anions could also intercalate into the MXene interlayer due to electrostatic interactions (Z*ifeng L. et al., Electrochemistry Communications, 2016, 72, 50-53.*). To test the intercalation of anions in this study, the concentration of both cations and anions in the permeate compartment was measured, which suggested that the concentrations increased stoichiometrically, consistent with previous studies regarding the ion-sieving property of 2D lamellar membranes (*Haihui W. et al., Nature Sustainability, 2020, 3, 296-302;* and *Joshi R. K. et al., Science, 2014, 343,752-754.)*. Based on this, we believe that both the cations and anions intercalated and then diffused through the membrane under concentration-driven conditions in this study. Moreover, as shown in the zeta potential measurement results (Fig. 2(i)), the surfaces of channels in the Mn-SAT membrane exhibited more negative polarity. Because of the different sensitivities of the high- and low-valent ions to electrostatic interactions with channel walls, the ions with various valences exhibited different permeation rates in the Mn-SAT membrane. Particularly, for NaCl and Na_2_SO_4_, due to the stronger electrostatic repulsion of the co-ions of SO_4_^2-^ against the channel walls when transported through the membrane, the transmembrane rate of divalent SO_4_^2-^ would be slower than that of monovalent Cl^−^. Considering the stoichiometric permeation constraint described above, NaCl exhibited a higher permeation rate than Na_2_SO_4_. Detailed supplemental experiments and discussions (Line 425, Page 19) have been added to address this issue in the revised manuscript and the Supplementary Materials as follows:

“For chloride salts and sulfate salts with the same cations, due to the stronger electrostatic repulsion of SO_4_^2-^ co-ions than Cl^−^ against the negatively charged channel wall when transported through the Mn-SAT membrane, divalent SO_4_^2−^ diffused slower than monovalent Cl^−^. For the electroneutrality constraint (Supplementary Fig. 19), the cations (Na^+^) and anions would both intercalate and diffuse through the membrane in a stoichiometric ratio, and NaCl exhibited a higher permeation rate than Na_2_SO_4_.

**Supplementary Figure 19. Concentrations of Na+ and Cl- in the permeate compartment through the Ca-SAT membrane with 0.5 M NaCl feed solution.** Cations and anions transport through the membrane in a stoichiometric manner to maintain the charge neutrality on each side, consistent with previous studies^3-4^.”

**Comment 6**
*Why is the intercalation/diffusion speed of Al3+ is that slow compared to all other ions despite the large interlayer spacing for the alginate-pillared membranes? The large interlayer spacing should significantly reduce the interaction and diffusion barrier for intercalation of high charge density Al3+ cations in the MXene membranes. This point needs a clear explanation, ideally supported by some experiments.*

**Response to Comment 6:**

We thank the reviewer for the kind comment. After the channels were pillared by the Ca-alginate gels, the effective diameter of the channels for ion transport in the membrane was fixed at 7.4±0.2 Å, which is comparable to the size of hydrated monovalent cations (for instance, Na^+^: 7.16 Å and K^+^: 6.62 Å) but smaller than most hydrated ions with a high valence (for instance, Mg2^+^: 8.56 Å and Al3^+^: 9.60 Å) (Supplementary Table 1). As described in previous reports regarding ion transport through extremely confined channels (*Thomas, M. et al., Small, 2014, 10, 1453-1465;* and *Richards, L. et al., Small, 2012, 8, 1701-1709.)*, the water molecules in the ion’s hydration shell would rearrange or even be partially stripped when an ion enters into an atomic-scale channel with sizes comparable to or smaller than the diameter of hydrated ions. The energy barrier for ion dehydration, which dominated in the dehydration-assisted transport process, is considered to be directly related to the magnitude of the ion’s hydration free energy (*Abraham, J. et al., Nature Nanotechnology, 2017, 12, 546-551.*). Although the hydration free energy depends on the electrostatic attraction between ions and polarized water molecules, the hydration free energy difference induced by ion valence is much more pronounced than that induced by ion radius. As summarized in Supplementary Table 1, the hydration energy of K^+^ (-435 kJ/mol) is only 100 kJ/mol higher than that of Na^+^ (-333 kJ/mol); however, the multivalent cations Mg^2+^ (-1868 kJ/mol) and Al^3+^ (-4665 kJ/mol) are approximately 1500 kJ/mol and 2700 kJ/mol higher than Na^+^, respectively. As a result, when they are ready to enter the stable nanochannel in the Ca-SAT membrane, alkali cations with weakly bound shells could experience a lower energy barrier and consequently exhibit a higher permeation rate, whereas Al^3+^, whose water shell was much ‘tougher’, needed to overcome a significant energy barrier to diffuse through the atom-scale channel and thus exhibited a significantly smaller diffusion rate than that of monovalent cations. We have added the explanation (Line 336, Page 16 and Line 352, Page 17) to clarify the statement in the manuscript as follows:

“As described above, the effective diameter of the nanochannel in the Ca-SAT membrane was fixed at 7.4±0.2 Å, which is comparable to the monovalent alkali cations (for instance, Na^+^: 7.16 Å and K^+^: 6.62 Å) but smaller than most of the hydrated ions with a high valence (for instance, Mg^2+^: 8.56 Å and Al^3+^: 9.60 Å) (Supplementary Table 1), and the increasing ion concentration with time in the permeate compartment, even at a low feed concentration, could verify ion dehydration during diffusion through the pillared membrane.

As a result, when hydrated cations are ready to enter the stable nanochannel in the Ca-SAT membrane, alkali cations could experience a lower energy barrier and consequently exhibit a higher PR. However, Al^3+^, whose water shell is much ‘tougher’ than the shell of other ions, needed to overcome a significant energy barrier to diffuse through the atom-scale channel, thereby exhibiting a significantly slower diffusion rate than that of the monovalent cations.”

**Comment 7**
*The authors do not discuss the effect of membrane thickness on the performance at all. That is an important parameter in the ion movement and interactions for MXene membranes. The authors need to show at least some experiment about this relation.*

**Response to Comment 7：**

We thank the reviewer for the kind suggestion. According to the reviewer’s suggestion, the thickness effect on the permeation rate of different cations has been investigated using Ca-SAT membranes with different thicknesses (1 µm, 3 µm, and 5 µm). The permeation rate exhibited an apparent increase with decreasing thickness, while the selectivity towards the valence of cations was barely influenced. The experimental data and discussion have been added in the revised manuscript (Line 233, Page 11) and the Supplementary Materials as below:

“We further studied the dependence of the cation PRs on the thickness of the Ca-SAT membrane (Supplementary Fig. 10). The ion PRs exhibited a distinct decrease with increasing thickness, which could be attributed to the prolonged permeation length. Nevertheless, we also observed that the ion-sieving property of the Ca-SAT membrane was dependent on the cation valence and was almost unaffected by the thickness variation.

**Supplementary Figure 10. Effect of membrane thickness (1, 3 and 5 μm) on ion permeation behavior through the Ca-SAT membranes.** Membranes with various thicknesses were synthesized by changing the volume of filtrated nanosheet solutions.”

**Comment 8**

*The authors indicate that they dry their membrane at RT. However, it is known that MXene membranes do not completely dry at RT even under vacuum. Some of the interlayer water even stay in MXene films after drying at temperatures higher than 500 C. Considering this, how can they claim that different cation-pillars in their alginate hybrid membranes are the only factor affecting the d-spacing in their films? Each of those cations can have a different hydration shell. Also, the amount of the absorbed water (interlayer water) is known to be higher for multivalent cations such as Mg2+ and Al3+.*

**Response to Comment 8：**

We thank the reviewer for the kind comment. We agree with the points indicated by the reviewer that the lamellar structure of the Ti_3_C_2_T*_x_* membrane is sensitive to the water molecules in the interlayer spacing, and these water molecules could not be completely removed when dried at RT. However, because this study is mainly concerned with the ion-sieving property of membranes under aqueous environments, after drying under the same conditions at RT, we confirmed only whether the structural variation induced by the intercalation of both water and cations in the aqueous solution was suppressed; and a test of the structural characteristics at different temperatures in atmospheric environments was not conducted. Additionally, we also agree with the reviewer that cations with hydration shells of different structures have a significant impact on the final d-spacing after intercalation. To suppress this structural variation and stabilize the interlayer spacing, we designed the hybridization structure by incorporating the hydrogel pillars into the interlayer spacing. Hydrogel pillars were formed in the crosslinking reactions between alginate molecules and multivalent cations, including Ca^2+^, Ba^2+^, Mn^2+^ and Al^3+^, in this study. As a result, unlike the cations that were spontaneously intercalated into the Ti_3_C_2_T*_x_* membrane, the multivalent cations in the SA-Ti_3_C_2_T*_x_* membrane were crosslinked with alginate sodium molecules. XPS was also conducted to confirm the existence of multivalent cations, and the Ca 2p spectrum was consistent with typical Ca-alginate gels (*Zhang M. J. et al., Water Research, 2018, 129, 337-346;* and *Guo Z. W. et al., Journal of Colloid and Interface Science, 2020, 562, 224-234.)*, which confirmed the crosslinking interactions between Ca^2+^ and alginate molecules. To make this clearer, we have added the discussion and XPS measurements in the revised manuscript (Line 180, Page 9 and Line 280, Page 13) and the Supplementary Materials as follows:

“Previous reports have confirmed that cations (and not anions) from aqueous solutions could spontaneously intercalate into the lamellar MXene membrane^37-38^, and the structural change depends on both the electrostatic attraction between the negatively charged Ti_3_C_2_T*_x_* sheets and the intercalated cations as well as the steric effect induced by the insertion of hydrated cations with different hydration shell structures. To stabilize the lamellar structure, hydrogel pillars that could form in the interlayer spacing during the crosslinking reaction between multivalent cations (including Ca^2+^, Ba^2+^, Mn^2+^ and Al^3+^ herein) and alginate molecules were incorporated in this study. As shown in Fig. 3 (c) and (d), the interlayer spacing of the Ca-SAT membrane is stably and effectively constrained at 16.2±0.2 Å, and both structural expansion and contraction are suppressed.

To further confirm the variation in the forms of Ca that existed and the concentration in the Ca-SAT membrane after ion permeation, XPS measurements were conducted. Consistent with the typical XPS spectrum of Ca-alginate gel in previous reports,^48-49^ the Ca 2p region could be fitted by two peaks at binding energies of 350.6 and 347.0 eV, which suggested the crosslinking interaction between Ca^2+^ and alginate molecules (Supplementary Fig. 15).

**Ref. 48:** Zhang, M. J.; Hong, H. C.; Lin, H. J.; Shen, L. G.; Yu, H. Y.; Ma, G. C.; Chen, J. R.; Liao, B. Q. Mechanistic insights into alginate fouling caused by calcium ions based on terahertz time-domain spectra analyses and DFT calculations. Water Res. 2018, 129, 337-346.

**Ref. 49:** Guo, Z. W.; Li, Q.; Li, Z. Y.; Liu, C.; Liu, X. R.; Liu, Y. Y.; Dong, G. L.; Lan, T.; Wei, Y. Fabrication of efficient alginate composite beads embedded with N-doped carbon dots and their application for enhanced rare earth elements adsorption from aqueous solutions. J. Colloid Interface Sci., 2020, 562, 224-234.

**Supplementary Figure 15. XPS analysis of Ca in the Ca-SAT membrane before (a) and after (b) the 0.5 M NaCl permeation test.** After the permeation test, the membrane was rinsed thoroughly with DI water until the conductivity of the rinse liquid was below 0.3 μS/cm to eliminate the disturbance from impurities before being subjected to XPS measurements.”

**Comment 9**

*A concerning point to me is about the MXene solutions the authors have made. In Figure SI 1, The UV-Vis spectra of the as prepared MXene solution is shown, which clearly shows that the Ti3C2Tx solution is highly oxidized. The broad bump at around 300 nm range are for TiO2. And from the intensity of the TiO2 peak in the overall MXene solution spectra I believe the solution authors have prepared is highly oxidized. That can have direct affect in hybridization process with alginate, as well as the performance of the membranes. I recommend either authors explain the reason for this severe oxidation in their solutions, its affect on the membranes and their processing, or try to process a new solution and suppress the oxidation of their MXenes. A normal Ti3C2Tx solution generally does not show the TiO2 bump or it should be very negligible. The minimum thing the authors must do is to show data for a non-oxidized MXene, and its film to prove either that its ion-sieving properties are similar to the oxidized, or different.*

**Response to Comment 9:**

We thank the reviewer for the kind comment. We agree with the reviewer that the oxidation of the Ti_3_C_2_T*_x_* colloid solution is indeed a critical factor during Ti_3_C_2_T*_x_* membrane preparation. We have reviewed the UV-vis measurement results of the Ti_3_C_2_T*_x_* dispersion in the previous literature (*Gogotsi Y. et al., Chemistry of Materials, 2017, 29, 7633-7644; Gogotsi Y. et al., Chemistry of Materials, 2017, 29, 4848-4856; Gogotsi Y. et al., ACS Applied Materials & Interfaces, 2018, 10, 24491-24498; Jingjing J. et al., Flatchem, 17, 2019,100128;* and *Satheeshkumar E. et al., Chemistry an Asian Journal, 2020, 15: 1044-1051.*). In particular, there are two representative studies conducted by Gogotsi’s group specific to the guidelines for the Ti_3_C_2_T*_x_* synthesis and oxidation stability of colloidal Ti_3_C_2_T*_x_* (*Gogotsi Y. et al., Chemistry of Materials, 2017, 29, 7633-7644;* and *Gogotsi Y. et al., Chemistry of Materials, 2017, 29, 4848-4856.*). We found that all the reported UV-vis spectra of the as-prepared Ti_3_C_2_T*_x_* dispersions in the literature contained similar broad absorption peaks within the range of 220-325 nm. Furthermore, a previous study (*Gogotsi Y. et al., ACS Applied Materials & Interfaces, 2018, 10, 24491-24498.*) also indicated that the shape of the bump around the 300 nm region in the UV-vis spectrum is dependent on the lateral size of the nanosheet. Prepared according to the same minimally intensive layer delamination (MILD) method, the spectra of large-size Ti_3_C_2_T*_x_* nanosheets showed a spectrum shape that was approximately identical to our results. Moreover, to further confirm the oxidation of Ti_3_C_2_T*_x_* nanosheets, UV-vis measurements were conducted again for fresh Ti_3_C_2_T*_x_* dispersions with different aging times at RT. We have confirmed that the intensity of the absorption peak at 750 nm in the UV-vis spectra, which was usually chosen as the metric for the concentration of the Ti_3_C_2_T*_x_* nanosheets, exhibited a distinct decrease over time; simultaneously, the broad absorption within the range of 220-325 nm also showed a clear shift to the left over time. A detailed discussion has been added to the Supplementary Materials as follows:

“**Supplementary Figure 1. Stability of the Ti_3_C_2_T*_x_* nanosheet dispersion stored at RT (a) Optical images of the dispersion changes over time (b) Normalized UV-vis spectra of the Ti_3_C_2_T*_x_* dispersion with different aging times.** We observed that the color of the Ti_3_C_2_T*_x_* solution changed from dark green to transparent as it underwent degradation. Correspondingly, in the UV-vis spectra, the intensity of the absorption peak at 750 nm, which was usually chosen as the metric for the concentration of the Ti_3_C_2_T*_x_* nanosheets, exhibited a distinct decrease over time, and the broad absorption bump within the range of 220-325 nm also showed a clear shift to the left over time^1-2^.

**Ref** 1: Zhang, C. F. J.; Pinilla, S.; McEvoy, N.; Cullen, C. P.; Anasori, B.; Long, E.; Park, S.; Seral-Ascaso, A.; Shmeliov, A.; Krishnan, D.; Morant, C.; Liu, X. H.; Duesberg, G. S.; Gogotsi, Y.; Nicolosi, V. Oxidation Stability of Colloidal Two-Dimensional Titanium Carbides (MXenes). Chem. Mater. 2017, 29, 4848-4856.

**Ref** 2: Alhabeb, M.; Maleski, K.; Anasori, P.; Lelyukh, P.; Clark, L.; Sin, S.; Gogotsi, Y. Guidelines for Synthesis and Processing of Two-Dimensional Titanium Carbide (Ti_3_C_2_T*_x_* MXene). Chem. Mater. 2017, 29, 7633−7644.”

**Reviewer comments, second version:**

Reviewer #2 (Remarks to the Author):

In this work, Ti3C2Tx membrane laminar architecture was effectively stabilized by various alginate hydrogel pillars and used for ion sieving. After the revision, the authors addressed all my comments and the manuscript is good for publication. The following minor points are recommended to be addressed.

1. The abstract is too long, near 300 words, which is difficult for the reader to grasp key points.
2. There are some prior published works using MXene for ion sieving. Please elaborate more on the novelty of this work in the Introduction part.

**Author rebuttal, second version:**

**Reviewer #2**

**General Comment**
*In this work, Ti3C2Tx membrane laminar architecture was effectively stabilized by various alginate hydrogel pillars and used for ion sieving. After the revision, the authors addressed all my comments and the manuscript is good for publication. The following minor points are recommended to be addressed.*
**Response to the General Comment**

We greatly appreciate the reviewer for the positive evaluation of our work.

**Comment 1**

*The abstract is too long, near 300 words, which is difficult for the reader to grasp key points.*

**Response to Comment 1**

We thank the reviewer for the kind suggestion. We have deleted the unnecessary description in the abstract and revised the statement (Line 18, Page 2) in the manuscript as follows:

“Two-dimensional membranes have attracted extensive interest due to the anomalous transport phenomena; however, the ions separation performance is below the theoretically prediction. The stabilization of the d-spacing is the key step for enhancing the ion selectivity. Here, we demonstrate a strategy for stabilizing the Ti_3_C_2_T*_x_* laminar architecture by alginate hydrogel pillars. After pillared by Ca-alginate, the nanochannel diameters are effectively fixed at 7.4±0.2 Å, and the membrane presents a permeation cutoff and an outstanding sieving property towards valent cations. When applied for acid recovery, the outstanding H^+^/Fe^2+^ selectivity makes the membrane a promising substitution for traditional ion-exchange membranes. Moreover, the ultrathin Mn-alginate pillared membrane with identical d-spacing exhibits a 100% Na_2_SO_4_ rejection with high water permeance, which is superior to the state-of-the-art nanofiltration membranes. Building on these findings, we demonstrate an efficient method to tune the ion selectivity and introduce a new perspective for energy- and environment-related applications.”

**Comment 2**

*There are some prior published works using MXene for ion sieving. Please elaborate more on the novelty of this work in the Introduction part.*

**Response to Comment 2**

We thank the reviewer for the kind reminder. We have cited the recent work and revised the explanation in the revised manuscript (Line 81, Page 4) as follows:

“A number of alternative strategies have been proposed to reduce the interlayer spacing, such as physical confinement, cation intercalation and self-crosslinking, leading to a high rejection of ions, independent of size or valence^12, 32, 39-40^. These membranes demonstrated the exclusion ability to the monovalent ions and hold the promise for desalination. Several studies have also been conducted to enhance the membrane structure stability by covalent crosslinking by polymer molecules; however, due to the volume effect induced by the crosslinking reactions, the membranes showed an improved molecular separation property but a decreased selectivity towards ions.^40-43^ ~~Therefore, how to effectively and precisely stabilize the d-spacing of the lamellar membrane to be comparable to~~ *~~D~~* ~~for achieving an accurate sieving performance in terms of the ion charge or size is still a technical challenge.~~ Therefore, how to effectively and precisely stabilize the d-spacing of the lamellar membrane to be comparable to hydrated ions diameter, and further achieve an accurate sieving performance in terms of the ion (cation or anion) charge or size is still a technical challenge.

**Ref 40**: Ding, L. et al. Effective ion sieving with Ti_3_C_2_T*_x_* MXene membranes for production of drinking water from seawater. *Nat. Sustain.* **3,** 296-302 (2020).”

**Reviewer comments, third version:**

**Author rebuttal, third version:**
